# An exhaustive cell-based screen coupled with an intracellular-induced lux-based reporter identified bioactive molecules that inhibit host cell infection by intracellular pathogens

**DOI:** 10.3389/fcimb.2026.1770677

**Published:** 2026-03-09

**Authors:** Boaz Adani, Alexander Plotnikov, Lena Lueken, Inna Shomer, Khriesto Shurrush, Nele Meyer, Katrin Künnemann, Malte Kellermann, David Margulies, Guntram A. Grassl, Michael Hensel, Haim Michael Barr, Ohad Gal-Mor

**Affiliations:** 1 The Infectious Diseases Research Laboratory, Sheba Medical Center, Tel-Hashomer, Israel; 2 Wohl Institute for Drug Discovery of the Nancy and Stephen Grand Israel National Center for Personalized Medicine, Weizmann Institute of Science, Rehovot, Israel; 3 Abt. Mikrobiologie, Universität Osnabrück, Osnabrück, Germany; 4 Institute of Medical Microbiology and Hospital Epidemiology, Hannover Medical School and German Center for Infection Research (DZIF), Hannover, Germany; 5 Department of Chemical and Structural Biology, Weizmann Institute of Science, Rehovot, Israel; 6 CellNanOs – Center of Cellular Nanoanalytics Osnabrück, Universität Osnabrück, Osnabrück, Germany; 7 Department of Clinical Microbiology and Immunology, Gray Faculty of Medical and Health Sciences, Tel-Aviv University, Tel-Aviv, Israel

**Keywords:** high-throughput screening (HTS), host-pathogen interactions, intracellular pathogens, intracellular replication, *Listeria monocytogenes*, *Salmonella enterica*, medicinal chemistry

## Abstract

**Introduction:**

Antibiotic resistance poses a critical and escalating global health crisis, leading to higher morbidity and mortality associated with infectious diseases. This problem is significantly exacerbated by intracellular bacterial pathogens, which are often shielded from conventional antibiotics and foster the emergence of persister populations. Recently, host-directed therapy (HDT) has been emerging as a promising strategy that aims to modulate host cellular processes or immune responses to enhance bacterial clearance. Nonetheless, the inherent complexity of host biology makes identifying appropriate and safe modulators challenging, unpredictable, and highly complicated.

**Methods:**

Here, we present a cell-based high-throughput screen (HTS), coupled with an intracellular-induced reporter that was used to screen a library of nearly 37,000 small molecules with potentially pharmacological activity for compounds that inhibit host cell infection by intracellular pathogens.

**Results and discussion:**

This multistage, screening protocol resulted in the identification of eight non-cytotoxic compounds that efficiently inhibited the intracellular growth of the Gram-negative bacterium *Salmonella* Typhimurium in human epithelial cells by ~2.5- to 6-fold, without inhibiting *Salmonella* growth in culture. Five of these eight molecules were also effective in controlling the intracellular replication of *Salmonella* in primary mouse macrophages by 1.5- to 38-fold. Strikingly, seven hits also inhibited the intracellular growth of the Gram-positive bacterial pathogen *Listeria monocytogenes* in epithelial cells by 1.5- to 10-fold. The structure–activity relationship approach successfully identified chemical analogs of one hit with enhanced biological activity as infection inhibitors. Overall, we describe a robust HTS platform that can be adapted for screening of compound libraries against other pathogens, and suggest that the identified compounds are potential candidates for downstream development of novel drugs against intracellular bacterial infections.

## Introduction

Infectious diseases pose an increasing critical global threat to both human and animal populations. A recent study estimated 4.71 million deaths associated with antibacterial-resistant infections annually and forecasted over 39 million deaths from antibiotic-resistant infections by 2050 (Collaborators and G.B.D.A.R (2024)). Even nowadays, many infections are difficult to treat, resulting in high-dose administration of antibacterials, unbearable antibiotic toxicity, delays in effective treatment, and increased mortality due to multidrug-resistant (MDR) infections (Kamaruzzaman et al., 2017).

Importantly, this challenge is specifically prominent in the context of infections caused by intracellular bacterial pathogens. These bacteria, including the genera *Salmonella*, *Listeria*, *Brucella*, *Rickettsia*, *Legionella*, *Chlamydia*, and *Mycobacterium*, invade host cells to establish an adapted replication niche, which facilitates their survival and dissemination. The intracellular lifestyle of these bacteria offers protection from the host’s humoral immunity, sequestration from neutrophils, and access to nutrients that may be scarce extracellularly (Petit and Lebreton, 2022; Li et al., 2023). As a result, intracellular bacterial pathogens have evolved to manipulate host cells to access their preferred niches within targeted cells. After invasion, bacteria are contained within a plasma membrane-derived vacuole, such as phagosomes or endosomes. Vacuolar bacteria, like *Salmonella enterica* (*S. enterica*) or *Mycobacterium tuberculosis* (*M. tuberculosis*), remain within the modified vacuoles, while cytosolic bacteria, like *Listeria monocytogenes* (*L. monocytogenes*), rupture the vacuole and reside within the host cytosol (Johnson et al., 2018; Gal-Mor, 2019; Petit and Lebreton, 2022; Li et al., 2023).

Intracellular pathogens have co-evolved with hosts and developed a striking ability to manipulate and subvert multiple host functions and pathways to facilitate their survival and transmission. In general, the process by which intracellular bacteria hijack host cells can be divided into four distinct stages: adhesion, internalization, survival/proliferation, and dissemination. Interfering with each one of these steps can help in controlling the infection and may be considered as a desired drug target.

*Salmonella enterica*, a ubiquitous foodborne pathogen, employs a sophisticated multistage strategy for host colonization and systemic dissemination, critically orchestrated by its type III secretion systems (T3SSs). Initial infection hinges on adhesion to host epithelial cells, followed by a rapid invasion of these non-phagocytic cells, a hallmark of *Salmonella*’s pathogenesis. This internalization is largely driven by the *Salmonella* pathogenicity island (SPI) 1-encoded T3SS, which translocates effector proteins to manipulate host actin dynamics, resulting in membrane ruffling and bacterial uptake (Fattinger et al., 2021). Once inside the cell, *Salmonella* establishes an intracellular niche, known as the *Salmonella*-containing vacuole (SCV), where it undergoes intracellular replication. This crucial step is facilitated by the SPI-2-encoded T3SS, which delivers a different array of effectors that remodel the SCV, prevent lysosomal fusion, and acquire nutrients, allowing the bacteria to survive and multiply within macrophages and other phagocytic cells (Liss and Hensel, 2015). Ultimately, *Salmonella* can achieve dissemination to systemic sites, including the spleen and liver, often within these infected phagocytes, further contributing to systemic disease (Gal-Mor, 2019).

Although significant progress has been made during the last decades in our understanding of how intracellular pathogens interact with their hosts (Kellermann et al., 2021), this group of pathogens remains a major clinical concern worldwide, and the gained knowledge has not been translated into new therapeutic approaches yet. This challenge has become even more urgent with the increasing prevalence of MDR strains that are often linked to a more severe disease outcome, and more strains that are resistant continue to emerge worldwide (Alara and Alara, 2024). Alarmingly, some of these bacteria are responsible for clinically important and prevalent diseases, including tuberculosis (TB), chlamydial infections, listeriosis, and invasive salmonellosis (Liu et al., 2020). Hence, there is a pressing need to better understand how intracellular pathogens interact with their hosts and to develop new approaches to treat these infections.

A novel anti-infectious therapeutic approach called host-directed therapy (HDT) is based on the modulation of host response by pharmacologically active compounds and has recently been highlighted as a promising strategy to counteract the emergence of antimicrobial resistance. This therapeutic approach aims to augment protective innate or adaptive host response needed for pathogen control and/or to limit its pathogenicity (Thom and D’elia, 2024). Because traditional antibiotics directly target bacterial processes, they employ strong selective pressure, driving the evolution of antibiotic resistance mechanisms in the pathogen. In contrast, HDT bypasses the pathogen’s direct resistance mechanisms, creating a favorable strategy to combat existing drug-resistant strains and slow the emergence of new resistance. However, the inherent complexity of host biology and immune response makes identifying effective, broad-ranged, and safe modulators challenging, unpredictable, and vastly complicated.

Traditional high-throughput screening (HTS) campaigns, largely designed to identify direct antibacterial agents in axenic culture, often fail to address the distinct challenges of intracellular pathogens, such as their shielded intracellular niche and complex host–pathogen interactions. Existing cell-based screens, while valuable, frequently rely on endpoint assays or indirect readouts that lack the sensitivity, specificity, and throughput required to robustly identify compounds that selectively inhibit intracellular bacterial replication or modulate host responses without direct cytotoxicity.

Here, we used the clinically relevant Gram-negative pathogen *S.* Typhimurium as a prototypic model for facultative intracellular bacterial pathogens and developed a cell-based HTS protocol, coupled with an intracellular lux-based reporter system, which allowed effective screening of a small molecule library comprising nearly 37,000 compounds. Eight non-cytotoxic compounds [saracatinib (AZD0530), penbutolol, AW00794SC, PCM-0103431, Arry-380 analog, conivaptan, SPB03336SC, and indacaterol maleate] with no growth inhibition in culture were found to efficiently inhibit the intracellular growth of *S.* Typhimurium in host cells. Interestingly, seven of these molecules were also demonstrated to inhibit the intracellular growth of the Gram-positive pathogen *L. monocytogenes*. Moreover, the structure–activity relationship (SAR) approach was applied for one leading hit, and chemical analogs with even improved infection-inhibiting activity were identified. Overall, we describe a robust HTS platform that can be adapted for the screening of compound libraries against other intracellular bacteria and parasites, and suggest that the identified compounds can be used as potential candidates for the development of novel drugs to treat intracellular bacterial infections.

## Materials and methods

### Bacterial strains and growth conditions

All strains and plasmids used in this study are listed in **Supplementary Table 1**. *Salmonella enterica* serovar Typhimurium strain SL1344 and *Listeria monocytogenes* strain EDG were used as prototypical Gram-negative and Gram-positive intracellular bacteria, respectively. *Salmonella* and *Escherichia coli* strains carrying the P*ssek3::lux* and P*rpoD::lux* (see **Supplementary Table 1**) were grown in LB-Lennox broth, PCN minimal medium (Neidhardt et al., 1974) [80 mM of MOPS pH 7.4/80 mM of MES pH 5.8, 1/0.4 mM of phosphate, 10 mM of glucose, 4 mM of tricine, 100 μM of FeCl_3_, 376 μM of K_2_SO_4_, 50 mM of NaCl, 10 nM of Na_2_MoO_4_, 10 nM of NaSeO_3_, 4 nM of H_3_BO_3_, 0.3 mM of CoCl_2_, 0.1 mM of CuSO_4_, 0.8 mM of MnCl_2_, 0.1 mM of ZnSO_4_, 15 mM of NH_4_Cl, 1 mM of MgSO_4_, 10 μM of CaCl_2_] with histidine, or in N-minimal medium [80 mM of MES (pH 5.8), 5 mM of KCl, 7.5 mM of (NH_4_)SO_4_, 0.5 mM of K_2_SO_4_, 337 μM of K_2_HPO_4_/KH_2_PO_4_ (pH 7.4), 20 mM of MgCl_2_, 38 mM of glycerol, and 0.1% casamino acids] with aeration at 37°C supplemented with kanamycin (50 µg/mL) with shaking (250 rpm).

### Construction of bioluminescence reporters

To construct a bioluminescence reporter strain, the *S*. Typhimurium *sseK3* promoter region was PCR-amplified using the primers “F XhoI sseK3 Pro” and “R BamHI sseK3 Pro” (**Supplementary Table 2**). The obtained 221-bp fragment was then cloned using the restriction enzymes *Xho*I and *Bam*HI into the pCS26 vector that carries the *luxCDABE* operon of the bacterium *Photorhabdus luminescens* (Bjarnason et al., 2003; Aviv and Gal-Mor, 2018). The resulting construct, P*ssek3::lux*, was electroporated into *S*. Typhimurium SL1344-electrocompetent cells. *Salmonella* Typhimurium SL1344 and *E. coli* DH5 harboring the *lux* reporting constructs were grown in LB at 37°C with shaking (250 rpm) for 16 h and then washed and subcultured (1:100) into fresh LB or N-minimal medium. Growth curves of the reporter strains were obtained by growing the cultures in LB and N-minimal medium in a white 96-well flat-bottom plate (Greiner Bio-One, Kremsmünster, Austria) at 37°C under regular shaking. Absorbance at 600 nm and bioluminescence were determined using the Infinite 200 PRO M-Plex microplate reader (Tecan, Männedorf, Switzerland) and imaged using IVIS Lumina LT (PerkinElmer, Shelton, USA) after 5.5 h of growth.

### Bioluminescence inhibition assay

To evaluate whether the compounds influence the bioluminescence of the intracellular reporter, luminescent *S*. Typhimurium was added to a sterile 96-well, cell culture-treated, flat-bottom white microplate, containing the compounds. An overnight culture of *S*. Typhimurium expressing P*sseK3::lux* was grown in PCN medium pH 7.4 with 1 mM of phosphate (non-luminescence-inducing medium) at 37°C, on a shaker incubator. Cells were sedimented at 13,000 rpm and washed with PCN medium pH 5.8 containing 0.4 mM of phosphate (luminescence-inducing medium) and used to inoculate a subculture 1:50 in PCN pH 5.8 with 0.4 mM of phosphate, grown for 6 h at 37°C on a shaker incubator. The subculture was diluted 1:20 with PBS, and luminescence induction was verified using the Tecan Infinite 200 PRO microplate reader. A total of 200 µL of the luminescent bacterial suspension was then added to each well of the microplate containing the compounds, and luminescence was measured directly, after 10 and 20 min. Bacterial suspension from a subculture grown in a non-luminescence-inducing medium was used as a reference control.

### Antibacterial assays

Overnight cultures of *S*. Typhimurium and *L. monocytogenes* were subcultured 1:100 in liquid LB and BHI medium, respectively, in a 96-well microplate in the presence and absence of the tested inhibitors and at a final concentration of 10 µM. Bacterial growth was followed for 24 h at 37°C with regular shaking by optical density reading at 600 nm using the Infinite 200 PRO M-Plex microplate reader (Tecan).

### Quantitative reverse transcription real-time PCR

*Salmonella* Typhimurium SL1344 was subcultured 1:100 into fresh LB medium in the presence of 10 µM compounds C2 (PCM-0086166) and C3 (PCM-0004846) and grown for 3 h to the late logarithmic phase (OD_600_ ~ 1.0). RNA was fixed using the RNAprotect Bacteria Reagent (QIAGEN) and extracted using the RNeasy Mini Kit (QIAGEN), according to the manufacturer’s instructions. Quantitative reverse transcription real-time PCR (qRT-PCR) was conducted as previously described (Elhadad et al., 2016), with a few modifications. Briefly, cDNA was synthesized using the qScript cDNA Synthesis Kit (Quantabio) using a T100 thermal cycler (Bio-Rad). Each reaction in a final volume of 20 μL was conducted in a 96-well optical reaction plate (Applied Biosystems) containing 10 μL of FastStart Universal SYBR green Master mix, 2 μL of cDNA, and target gene-specific primers (**Supplementary Table 2**) at a final concentration of 0.3 μM each. Melting curve analysis was applied to verify that each qRT-PCR reaction has generated a single amplimer. Relative quantification of transcripts was determined using the comparative threshold cycle (CT) method (Livak and Schmittgen, 2001), while transcript levels were normalized to the housekeeping gene *rpoD*. The Δ*CT* values were calculated by determining the difference in threshold values for the target gene and *rpoD* in cultures grown in the presence of compounds versus the threshold values of cultures grown in the absence of compounds (LB broth). The ΔΔ*CT* value was calculated by subtracting the normalized Δ*CT* value obtained for the LB culture from the normalized Δ*CT* value obtained for the culture grown with the compounds.

### Small molecule library

A total of 36,855 compounds (**Supplementary Table 3**) from the Weizmann Institute screening collection were included in this study. The compounds were compiled from several commercial screening collections (Sigma LOPAC, Prestwick Known Drugs, MicroSource-Spectrum, Selleck Bioactive, Maybridge HitFinder, Enamine, and ChemDiv). Compounds were dissolved to 10 mM stocks in dimethyl sulfoxide (DMSO) and stored in a desiccated nitrogen-flushed environment until use.

### High-throughput screening

The molecule library screen was performed in sterile tissue culture-treated white 384 flat-bottom well plates (Greiner) preloaded with 36,855 small molecules, at a final concentration of 10 µM using the Echo555 acoustic transfer system (Labcyte, Germany). Plates were seeded with HeLa cells suspended in DMEM containing 10% FCS (8 × 10^3^ cells/well) using the Multidrop Combi Reagent Dispenser (Thermo Fisher Scientific, Waltham, USA) and incubated at 37°C in 5% CO_2_ humidified incubator for 4 h to allow the cells to adhere. Each plate included 16 wells of 2 μM of cytochalasin D (CD) that was used as a positive control. An overnight culture of *S*. Typhimurium expressing P*sseK3::lux* was subcultured 1:100 in LB medium and grown for 3 h at 37°C with shaking (250 rpm) to the late logarithmic phase (OD_600_ ~ 1). The cultures were then diluted 1:50, and aliquots of 25 µL were used to infect the cells at a multiplicity of infection (MOI) of 1:50 (cell to bacteria). One hour post-infection (hpi), gentamicin at a final concentration of 20 µg/mL was added to each well to eliminate extracellular bacteria, and the plates were incubated overnight (˜16 h) at 37°C under 5% CO_2_ atmosphere in a LiCONiC tissue culture incubator (LiCONiC Instruments, North Reading, USA). The next day, the infected cells were washed with phosphate-buffered saline (PBS), using a BioTek EL406 washer dispenser (BioTek Instruments, Winooski, USA), and the bioluminescence of the cells was detected by a LUMIstar Omega Plate Reader (BMG Labtech, Ortenberg, Germany), a rapid plate reader at detection mode: luminescence, a gain of 3,700, integration time of 200 ms, and plate chamber temperature of 37°C. Genedata Screener (Basel, Switzerland) was used for the normalization and interpretation of results.

### Epithelial cell infection with *Salmonella* and *Listeria*

To evaluate the infection inhibition activity of the compounds using direct bacterial counting, 5 × 10^4^ HeLa cells were suspended in DMEM (+10% FCS), seeded in a 24-well, cell culture-treated, flat-bottom microplate (Greiner), and incubated at 37°C in a humidified 5% CO_2_ incubator overnight prior to infection. The following day, the medium was replaced with fresh DMEM (+10% FCS) medium containing the PCM-0094889, PCM-0086166, PCM-0004846, PCM-0103431, PCM-0095494, PCM-0095293, PCM-0001349, and PCM-0095564 compounds at a final concentration of 10 µM, which were incubated with the cells for 4 h before the infection. An overnight *S*. Typhimurium SL1344 culture was subcultured 1:100 into fresh LB medium and grown at 37°C on a shaker incubator to the late logarithmic phase. After 3 h, the bacteria were diluted 1:100 in DMEM, and 500 µL from the diluted bacteria were added into each well at an MOI of 1:50. The infected cells were spun down for 5 min at 1,000*g* and then incubated at 37°C under 5% CO_2_ atmosphere. After 10 min, cells were washed three times with PBS, and fresh DMEM medium supplemented with 10 µM of each compound was added to each well and incubated for an additional 20 min. At 30 min post-infection, the medium was replaced with a fresh medium containing 10 µM of each compound and 100 µg/mL of gentamicin and incubated for 90 min. At 2 hpi, cells were washed three times with PBS and lysed with 250 µL of lysis buffer (0.1% SDS, 1% Triton X-100 in PBS) by incubating the cells for 10 min at room temperature with gentle agitation. To calculate the number of invading bacteria at 2 hpi, the cell lysates were serially diluted in PBS and plated onto LB agar plates for CFU count. To calculate the intracellular fold replication, at 2 hpi, the medium of the infected cells was replaced with DMEM supplemented with 10 µM of each compound and 10 µg/mL of gentamicin and incubated at 37°C in a humidified 5% CO_2_ incubator. At 24 hpi, the cells were washed three times with PBS and lysed with 250 µL of lysis buffer as above. Intracellular replication was determined by the ratio between the number of intracellular bacteria (CFU) counted at 24 hpi and their number at 2 hpi.

To infect HeLa cells with *L. monocytogenes*, cells were prepared the same way and incubated for 3 h with fresh DMEM (+10% FCS) supplemented with the tested compounds at a final concentration of 10 µM. Overnight cultures of *L. monocytogenes* that were grown statically in BHI at 30°C were washed and resuspended in PBS. Cell infection at an MOI of 1:50 was achieved by adding 20 µL of *Listeria* suspension to each well, followed by incubation at 37°C under 5% CO_2_ atmosphere. Thirty minutes post-infection, the cells were washed with PBS, and the medium was replaced with fresh medium supplemented with 10 µM of the tested compounds. One hpi, gentamicin was added to each well at a final concentration of 50 µg/mL. At 6 hpi, the cells were washed with PBS and lysed with 250 µL of DDW at room temperature. The cell lysates were then diluted in PBS and plated on LB-agar plates for CFU count. *Listeria* infection was calculated as the number of intracellular bacteria recovered at 6 hpi, divided by the infecting inoculum (CFUs).

For infection of HT29 cells, 3 × 10^5^ HT29-MTX-E12 cells per well were seeded in a 24-well plate. The day after seeding, cells were treated with the inhibitors at the indicated concentrations. Three hours later, cells were infected with a mid-logarithmic culture of *S.* Typhimurium SL1344 at an MOI of 30. After 30 min, cells were washed three times, and medium containing inhibitors and gentamicin (100 µg/mL) was added. One hour later, the medium was removed and replaced with the medium containing inhibitors and gentamicin (10 µg/mL). Twenty-four hpi, cells were washed three times with PBS and lysed with 500 µL of lysis buffer (0.1% SDS, 1% Triton X-100 in PBS) for 10 min at room temperature. Serial dilutions were plated on LB agar plates for CFU counting.

### Dose–response relationship assay in HeLa cells

To evaluate the inhibition activity of the compounds in a dose–response assay, 4.6 × 10^4^ HeLa cells were seeded in each well of a sterile 96-well, cell culture-treated, flat-bottom white microplate. Cells were incubated at 37°C/5% CO_2_ humidified incubator for 4 h in the presence of 0, 0.25, 1, 3.25, 10, and 30 µM of compounds PCM-0094889, PCM-0086166, PCM-0004846, PCM-0103431, PCM-0095494, PCM-0095293, PCM-0001349, and PCM-0095564. An overnight *S*. Typhimurium culture expressing P*ssek3::lux* was subcultured 1:100 in LB medium for 3 h at 37°C on a shaker incubator to the late logarithmic phase (OD_600_ ~ 1). The *Salmonella* culture was then diluted 1:50 in DMEM, and aliquots of 100 µL were added to each well to reach an MOI of 1:30 (cells per bacteria) and incubated at 37°C, 5% CO_2_ to allow infection. One hpi, gentamicin was added to a final concentration of 20 µg/mL, and the plates were incubated overnight. The next day, the cells were washed three times with sterile PBS, and bioluminescence was read using the Infinite M-Plex Tecan plate reader.

### Bone marrow-derived macrophage infection

Bone marrow-derived macrophages (BMDMs) were isolated from the femur leg bone of 7-week-old SWISS female mice. Macrophages were diluted in BMDM medium (50% DMEM high glucose, 20% FCS, 30% L-929 conditioned medium, 2 mM of L-glutamine, 1 mM of sodium pyruvate, 50 nM of β-mercaptoethanol) and seeded at 2.5 × 10^5^ cells/ml in a 24-well, cell culture-treated dish, 24 h prior to *Salmonella* infection. The next day, the BMDM medium was replaced with fresh BMDM medium supplemented with 10 µM of the tested compounds and incubated for 3 h at 37°C under 5% CO_2_ atmosphere. Macrophages were infected at an MOI of 1:10 with cultures of *S*. Typhimurium SL1344 and its Δ*ssaR* isogenic mutant as a negative control. Infected cells were spun down at 1,000*g* for 5 min and incubated for 30 min at 37°C, 5% CO_2_. Then, cells were washed three times with PBS to remove extracellular bacteria, and BMDM medium containing 100 μg/mL of gentamicin was added for 1 h of incubation. Cells were then washed three times with PBS, and the medium was replaced with fresh BMDM containing 10 μg/mL of gentamicin. To determine the intracellular growth of *Salmonella* at 2 and 24 hpi, cells were washed three times with PBS and lysed with 250 μL of lysis buffer (0.1% SDS, 1% Triton X-100 in PBS). The number of intracellular CFUs was quantified by plating serial dilutions of cell lysates on selective (streptomycin) LB-agar plates. *Salmonella* uptake was calculated as the number of intracellular bacteria recovered at 2 hpi, divided by the infecting inoculum (CFUs). *Salmonella* survival was calculated as the number of intracellular bacteria recovered at 24 hpi, divided by the number of intracellular bacteria recovered at 2 hpi.

### Cytotoxicity assay

To examine the potential toxicity of the identified hits to host cells, the CellTiter-Glo (GTG) Luminescent Cell Viability Assay (Promega, Madison, USA) was used. Sterile tissue culture-treated white 384 flat-bottom well plates (Greiner) were loaded with compounds at final concentrations of 0.3, 1, 3, 10, and 30 µM using the Echo555 acoustic transfer system (Labcyte, Germany). HeLa (2 × 10^3^) or HB2 epithelial cells were suspended in DMEM containing 10% FCS, and 50 µL of cell suspension was added to each well using Multidrop (Thermo Fisher Scientific) and incubated for 5 h. Gentamicin at a final concentration of 20 µg/mL was added to each well, and the cells were incubated overnight. The CTG assay was performed in accordance with the manufacturer’s protocol. Briefly, the medium was aspirated, and 10 µL of the CTG reagent was added to each well. Then, the plates were shaken for 2 min followed by 10 min of incubation at 37°C under 5% CO_2_ atmosphere. Luminescent signal was detected using a PheraStar FS plate reader (BMG Labtech, Ortenberg, Germany). The results were analyzed using GeneData software (Basel, Switzerland).

### SAR studies

To implement a SAR study (Wawer and Bajorath, 2009), 41 commercially available close analogs of compound C4 (**Supplementary Table 5**) were obtained from Molport EU (www.molport.com) and tested for their ability to inhibit *S*. Typhimurium infection of human epithelial cells. To test the activity of the chemical analogs, 4.6 × 10^4^ HeLa cells were seeded in each well of a 96-well, cell culture-treated, flat-bottom white microplate (Greiner) and incubated at 37°C in 5% CO_2_ humidified incubator for 4 h, in the presence of 10 µM of compound PCM-0103431 and its chemical derivatives (**Supplementary Table 4**). Cells were infected with a subculture of *S*. Typhimurium expressing the reporter system P*ssek3::lux* as described above at an MOI of 1:50. One hpi, gentamicin was added to a final concentration of 20 µg/mL, and the infected cells were incubated overnight. The next day, the wells were washed three times with PBS, and bioluminescence was read using the Infinite 200 PRO M-Plex microplate reader (TECAN).

## Results

### Construction and functional characterization of a reporter system for intracellular *Salmonella* Typhimurium

**Figure 1:**
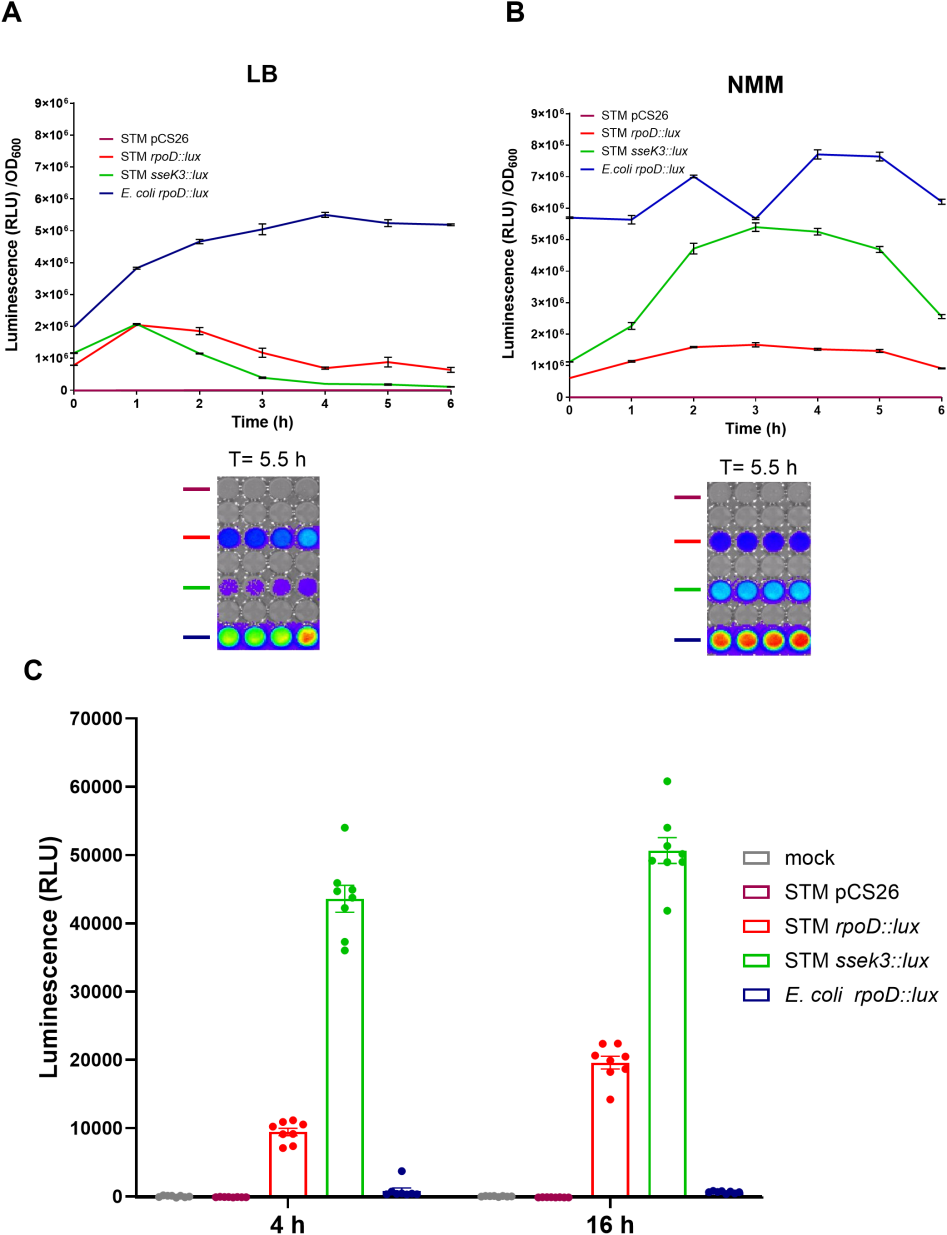
Functional characterization of a luminescence reporter system as a readout for intracellular *Salmonella* Typhimurium. *Salmonella* Typhimurium SL1344 harboring the empty autobioluminescent plasmid pCS26, P*ropD::lux*, or P*sseK3::lux* and *Escherichia coli* DH5 harboring P*ropD::lux* were subcultured into LB **(A)** or N-minimal medium **(B)** and grown for 6 h at 37°C under constant shaking. The optical density and the bioluminescence of the cultures were measured and expressed as the mean bioluminescence reading (of four replicates) normalized to their optical density at 600 nm (OD_600_). An image of the bioluminescence signal produced by these cultures was taken using the IVIS Lumina LT (PerkinElmer) imaging system and is shown after 5.5 h of growth from subculture. **(C)** HeLa cells were suspended in DMEM medium, seeded in white 96-well plate, and incubated at 37°C under 5% CO_2_ for 4 and 16 h prior to infection to allow the cells to adhere. Cells were then infected with *E. coli* expressing the P*rpoD::lux* and *S*. Typhimurium strains expressing either P*rpoD::lux* or P*ssek3::lux*. Non-internalized bacteria were killed by gentamicin, and at 16 h post-infection, bioluminescence was recorded and expressed in relative light units (RLU). Uninfected cells (mock) or cells infected with *S.* Typhimurium (STM) harboring the empty vector (pCS26) were used as negative controls.

To facilitate efficient screening of molecules that can potentially inhibit host cell infection by *S*. Typhimurium, we sought to construct a reliable reporter system that could be used in a quantitative HTS. Since bioluminescence generally provides a broader dynamic range and lower noise-to-signal ratio over fluorescence (Fan and Wood, 2007), we decided to use a self-sufficient bacterial luminescence system that does not require the addition of an external substrate (Aviv and Gal-Mor, 2018). For that purpose, we cloned the *S*. Typhimurium *rpoD* and *sseK3* promoters upstream to the *luxCDABE* operon of the bacterium *Photorhabdus luminescens* carried on the plasmid pCS26 (see **Supplementary Table 1**). RpoD (σ70) is the primary housekeeping sigma factor, responsible for the expression of *Salmonella* genes required for exponential growth under normal cellular conditions (Park et al., 2024), while SseK3 is a T3SS-2 translocated effector, whose expression is induced, while *Salmonella* is intracellular or under SPI-2-inducing conditions (Brown et al., 2011). **Figure 1** shows the bioluminescence signal of these reporter strains during growth in liquid culture in LB broth (**Figure 1A**) and in acidic N-minimal medium (NMM; pH 5.8; **Figure 1B**), considered as SPI-1- and SPI-2-inducing conditions, respectively.

While the *S*. Typhimurium strain expressing the P*rpoD::lux* reporter showed similar signal levels of luminescence in LB and NMM, the P*ssek3::lux* reporter was prominently induced in an *S*. Typhimurium strain grown in NMM and demonstrated high levels of luminescence under these conditions. Interestingly, an *E. coli* strain expressing the *lux* operon under an *S*. Typhimurium *rpoD* promoter also produced high levels of bioluminescence both in LB and NMM, indicating high and constitutive expression of this promoter in the *E. coli* background.

To test the ability of these strains to be used as a reporter system for intracellular bacteria, HeLa cells were infected with *E. coli* expressing the P*rpoD::lux* and *S*. Typhimurium strains expressing either the P*rpoD::lux* or P*ssek3::lux* reporters. Non-internalized bacteria were killed by the addition of gentamicin to the medium, and at 16 hpi, bioluminescence was examined as a readout for the intracellular bacterial load. HeLa cells infected with *S.* Typhimurium expressing the P*rpoD::lux* or P*ssek3::lux* constructs resulted in a high and specific bioluminescence signal that was readily detected in the infected host cells. Uninfected cells (mock) or cells infected with *S*. Typhimurium carrying the empty vector produced almost no luminescence, indicating a low background-to-signal ratio. Similarly, in contrast to the high signal obtained by the *E. coli* strain harboring the P*rpoD::lux* reporter extracellularly, this strain produced very low bioluminescence signal in the context of HeLa cell infection, due to its poor ability to invade these epithelial host cells (**Figure 1C**).

Because the signal of the P*sseK3::lux* reporter was higher than the signal of P*rpoD::lux* in HeLa cells infected with *S.* Typhimurium, and because similar results were obtained after 4 or 16 h of incubation host cells to allow sufficient cell adhesion prior to the infection, we decided to apply 4 h of incubation of host cells before infection with *S.* Typhimurium carrying the P*sseK3::lux* reporter in the subsequent HTS.

### A cell-based HTS successfully identified *Salmonella* infection inhibitors

**Figure 2:**
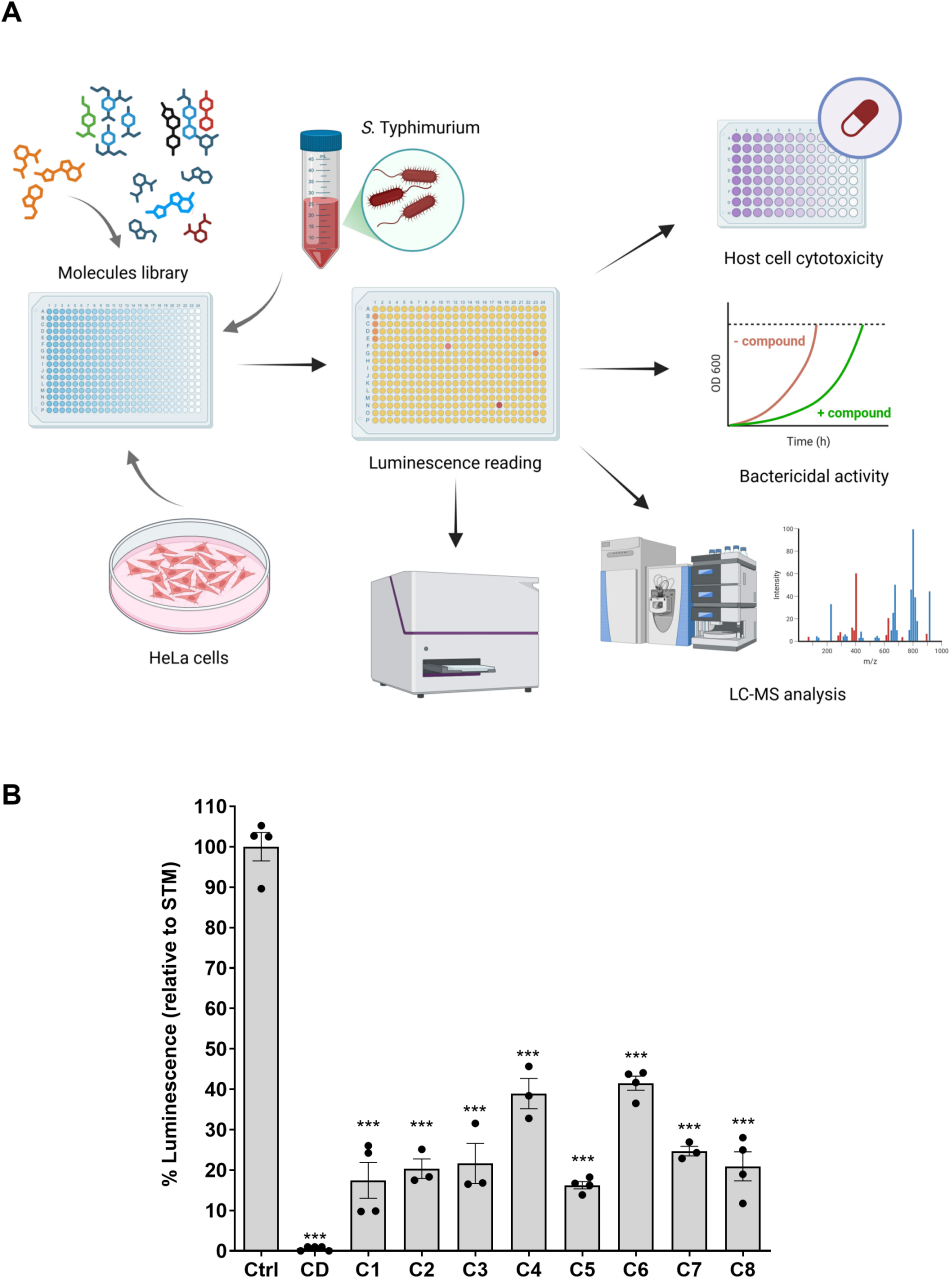
Cell-based high-throughput screening and identification of host cell infection inhibitors. **(A)** A schematic illustration of the pipeline used for the HTS. Infection assay of the intracellular pathogen *S*. Typhimurium carrying the reporter system was performed in HeLa cells in the presence of 36,855 bioactive molecules. Compounds that were found to reduce the bioluminescence signal (*N* = 473) were selected for the next stage. To filter out compounds that are cytotoxic to HeLa cells, the compounds were examined for cytotoxicity in HeLa cells at 10 µM concentration. Non-cytotoxic compounds (*N* = 175) continued to the next screening step. The antibacterial activity of the compounds was tested, and 101 compounds that showed antibacterial activity against *S*. Typhimurium were filtered out. Seventy-four qualified compounds were tested in a second independent test to confirm their ability to inhibit *Salmonella* infection. Hits that presented at least 50% decrease in the intracellular bioluminescence signal (*N* = 68) were selected for the next stage. Six out of 68 compounds that were found unstable in a liquid chromatography–mass spectrometry (LC–MS) analysis were excluded, and the remaining 62 compounds were tested for their ability to inhibit *Salmonella* infection in the dose–response infection experiments. At the end of the screen, we identified eight molecules that were able to inhibit *Salmonella* infection in HeLa cells and showed low cytotoxicity, no antibacterial activity, and high purity. **(B)** The ability of the eight compounds (C1 to C8) to inhibit *Salmonella* Typhimurium (STM) invasion into HeLa cells was determined by the bioluminescence assay as described in the *Materials and methods* section. *Salmonella* infection in the presence of the eight compounds at a final concentration of 10 µM is shown relative to its infection in the absence of the compounds (Ctrl). Cytochalasin D (CD) that blocks *Salmonella* invasion was used as a positive control. The bars show the mean and the standard error of the mean (SEM) of three to six biological repeats. One-way ANOVA was used to determine statistical significance. ***, P-value < 0.001.

The *S.* Typhimurium strain expressing the P*sseK3::lux* reporter was used in a seven-stage screening cascade, aiming to identify small molecules that can inhibit intracellular growth of *S.* Typhimurium. The different stages of the screening campaign and the number of compounds that passed each one of the filtering criteria are illustrated in **Figure 2A** and summarized in **Table 1**, respectively. To facilitate this HTS, HeLa cells were seeded in 384-well plates and incubated for 4 h in the presence of 36,855 compounds composing a collection of small molecules from different commercial libraries of putative bioactive molecules, at a final concentration of 10 µM (**Supplementary Table 3**). The incubated cells were then infected with the reporter *S*. Typhimurium P*sseK3::lux* strain for 1 h, washed, and incubated in the presence of gentamicin. Gentamicin is a potent bactericidal antibiotic that effectively kills extracellular *Salmonella*, but has poor penetration into eukaryotic host cells, and therefore, intracellular bacteria are “protected” from killing and survive this treatment. At 16 hpi, the bioluminescence signal in each well was determined by a plate reader and compared to the signal of cells that were incubated with 0.1% DMSO and infected with *S.* Typhimurium expressing P*sseK3::lux*, which was used as a reference control (Ctrl). As a positive control for a potent infection inhibitor, we included CD that blocks actin polymerization and therefore inhibits *Salmonella* invasion into host cells (Finlay et al., 1991). Since the intensity of the luminescence is generally proportional to the number of intracellular *Salmonella*, compounds that inhibit either the bacterial invasion or their intracellular replication are expected to result in a lower bioluminescence signal. Therefore, molecules that reduced the bioluminescence in the infected cells by 50% or more were selected as potential infection inhibitors for the next screening stage. During the first screening stage, we identified 473 (1.2% from all tested compounds) molecules that met this inclusion criterion.

**Table 1:** Different stages of HTS to identify *Salmonella* infection inhibitors.

Screening stage	Phenotype tested	Assay used	Inclusion criteria	Number of tested molecules	Number of qualified molecules	Number of technical repeats	Number of biological repeats
1	HeLa cell infection	Bioluminescence signal of intracellular bacteria	50% or more reduction in the bioluminescence intensity	36,855	473	1	1
2	Toxicity to HeLa cell	CellTiter-Glo Luminescent Cell Viability Assay	90% or more viability of HeLa cells following 16 h incubation	473	175	1	2
3	Antibacterial against *Salmonella*	Bacterial growth in LB by OD_600_ reading	No significant inhibition of *S*. Typhimurium growth	175	74	1	3
4	HeLa cell infection	Bioluminescence signal of intracellular bacteria	50% or more reduction in bioluminescence intensity	74	68	1	2
5	Chemical stability	Compounds purity by LC–MS analysis	Purity of 70% or more	68	62	1	1
6	No effect on bioluminescence production	Bioluminescence signal in bacterial culture	Reduction of ≤30% in luminescence	62	54	1	2
7	HeLa cell infection	Bioluminescence signal of intracellular bacteria in the presence of 0.25, 1, 3.25, 10, and 30 µM drug	Dose–response behavior	54	8	1	3

However, since a reduced signal can also result from cytotoxicity to HeLa cells or antibacterial activity against *Salmonella*, further filtering of candidates was applied in the next steps to eliminate compounds with an undesired cytotoxic mode of activity. To this end, we tested the cytotoxic activity of the 473 candidates at 10 µM concentration, using the CellTiter-Glo Luminescent Cell Viability Assay (Promega), and continued screening only compounds that maintained HeLa cell viability of 90% or more after incubation for 24 h in the presence of the compounds. Filtering out 298 compounds that did not meet this threshold resulted in 175 molecules that were qualified for the next screening stage.

In the following step, we sought to filter out compounds with antibacterial activity, as we wanted to avoid molecules that directly kill or inhibit bacterial growth, and focus only on those that can specifically inhibit host cell infection. To this end, we tested the ability of the 175 eligible compounds to inhibit the growth of subcultured *S*. Typhimurium in LB medium by following their optical density at 600 nm for 24 h. As expected, a significant portion of 101 out of the 175 compounds tested was found to impair *Salmonella* extracellular and cell-independent growth, and after their exclusion, we continued the screening with 74 non-cytotoxic molecules that showed no growth inhibition under the tested conditions. This group of molecules was tested again, in a second independent assay, for their ability to inhibit intracellular growth of *S*. Typhimurium, by following their bioluminescence signal in HeLa cells at 16 hpi. In this stage, 68 out of 74 tested compounds demonstrated again 50% or more reduction of the intracellular bioluminescence signal and were therefore selected for the next screening stage.

Subsequently, we tested the 68 selected compounds using liquid chromatography–mass spectrometry (LC–MS) to confirm the presence of >70% expected mass of these molecules. Six compounds that did not pass this criterion were excluded from the screen, resulting in a short list of 62 hits. Among this group of compounds, eight molecules were found to inhibit the bioluminescence production in *S.* Typhimurium culture that was grown in PCN pH 5.8 medium (Deiwick et al., 1999) as a *ssek3*-inducing medium (**Supplementary Table 4**) and, therefore, disqualified for downstream analysis. The remaining 54 compounds were next tested in a dose–response infection assay, using their bioluminescence as a readout for host cell infection. In these experiments, the ability of the 54 tested compounds to inhibit *Salmonella* intracellular growth in HeLa was studied under increasing drug concentrations of 0.25, 1, 3.25, 10, and 30 µM. Consequently, in this last stage of the screen, eight compounds (PCM-0094889, PCM-0086166, PCM-0004846, PCM-0103431, PCM-0095494, PCM-0095293, PCM-0001349, and PCM-0095564) that presented a clear dose–response behavior (**Supplementary Figure 1A**) were selected as the final candidates of the screen. For simplicity, these compounds will be called hereafter as C1 to C8 (**Table 2**). The inhibitory effect of these final eight compounds at 10 µM concentration on HeLa cell infection, by looking at the intracellular luminescence of the reporter *S*. Typhimurium strain, is shown in **Figures 2B**, and their cytotoxicity activity toward HeLa and HB2 epithelial cells is shown in **Supplementary Figure 1B**.

**Table 2:** Top 8 hits identified in the HTS.

#	PCM number	Molecule name	PCM structure	MW	Intracellular luminescence (%) relative to mock ± SE	HeLa viability (%)
C1	PCM-0094889	Saracatinib (AZD0530)	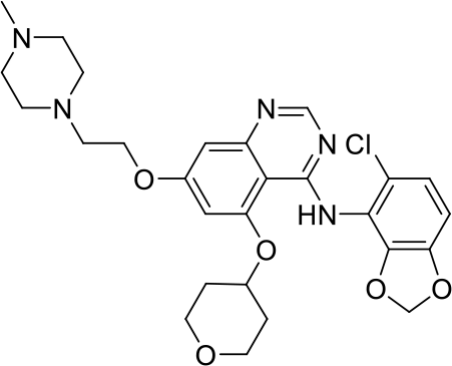	542.0	17 ± 4	96.0
C2	PCM-0086166	Penbutolol	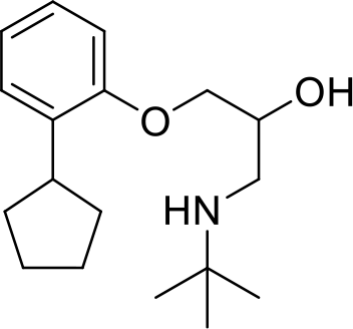	291.4	20 ± 2	100.0
C3	PCM-0004846	AW00794SC	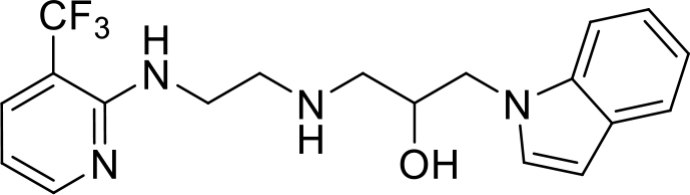	378.4	22 ± 5	91.2
C4	PCM-0103431		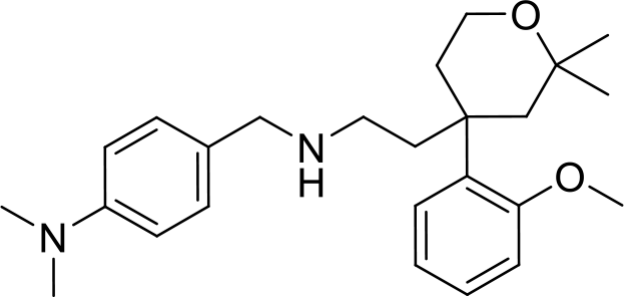	396.6	39 ± 4	91.0
C5	PCM-0095494	Arry-380 analog	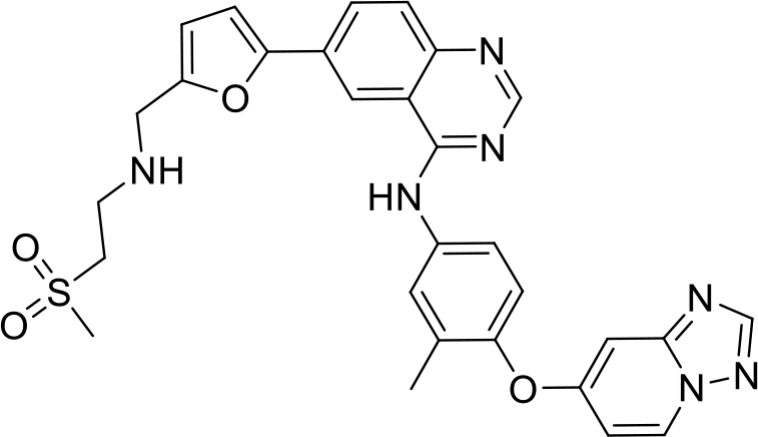	569.6	16 ± 1	99.7
C6	PCM-0095293	Conivaptan	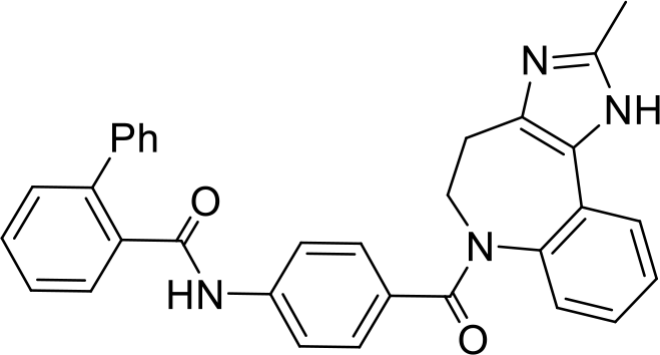	535.0	41 ± 2	104.9
C7	PCM-0001349	SPB03336SC	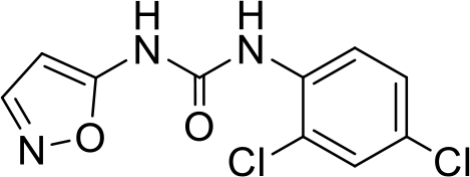	272.1	25 ± 1	93.8
C8	PCM-0095564	Indacaterol maleate	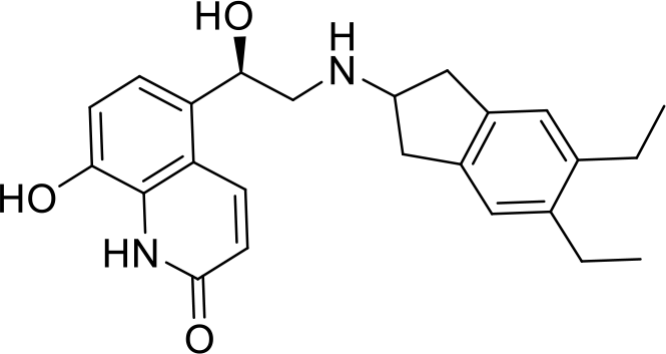	392.5	21 ± 4	101.5

Collectively, we concluded from these experiments that the final eight hits identified in the HTS have the potential to significantly inhibit either the invasion or intracellular replication of *S.* Typhimurium in epithelial host cells, and that at a concentration of 10 µM or less, these compounds are not cytotoxic to HeLa nor to HB2 cells.

Interestingly, five out of the eight compounds identified have known biological or pharmaceutical functions. Compound C1 (PCM-0094889), called saracatinib (AZD0530), is a tyrosine kinase inhibitor that primarily targets Src/Abl family kinases (Hennequin et al., 2006). Another putative tyrosine kinase inhibitor is C5 (PCM-0095494), which is annotated as an Arry-380 (AKA ONT-380 or tucatinib) analog inhibiting HER2. Compound C2 (PCM-0086166), penbutolol sulfate, is a beta-adrenergic receptor antagonist or beta-blocker. Compound C8 (PCM-0095564), indacaterol maleate, also belongs to the β-adrenergic receptor-targeting drug group. Compound C6 (PCM-0095293), conivaptan, is a dual arginine vasopressin (AVP) receptor antagonist that blocks both V1A and V2 receptors. Compound C3 (PCM-0004846) is a potential cholinesterase enzyme (AChE and BChE) inhibitor that was studied for its anti-Alzheimer properties (Ansari et al., 2024). Compounds C4 (PCM-0103431) and C7 (PCM-0001349) are not functionally characterized, and information about their biological function is unavailable. Nevertheless, the fact that compounds C1 and C5 are both inhibiting tyrosine kinases and that compounds C2 and C8 are beta-blockers suggests that the final hits may belong to several functional groups that target the same pathways.

### Identified hits inhibit intracellular replication of *Salmonella* in epithelial cells

**Figure 3:**
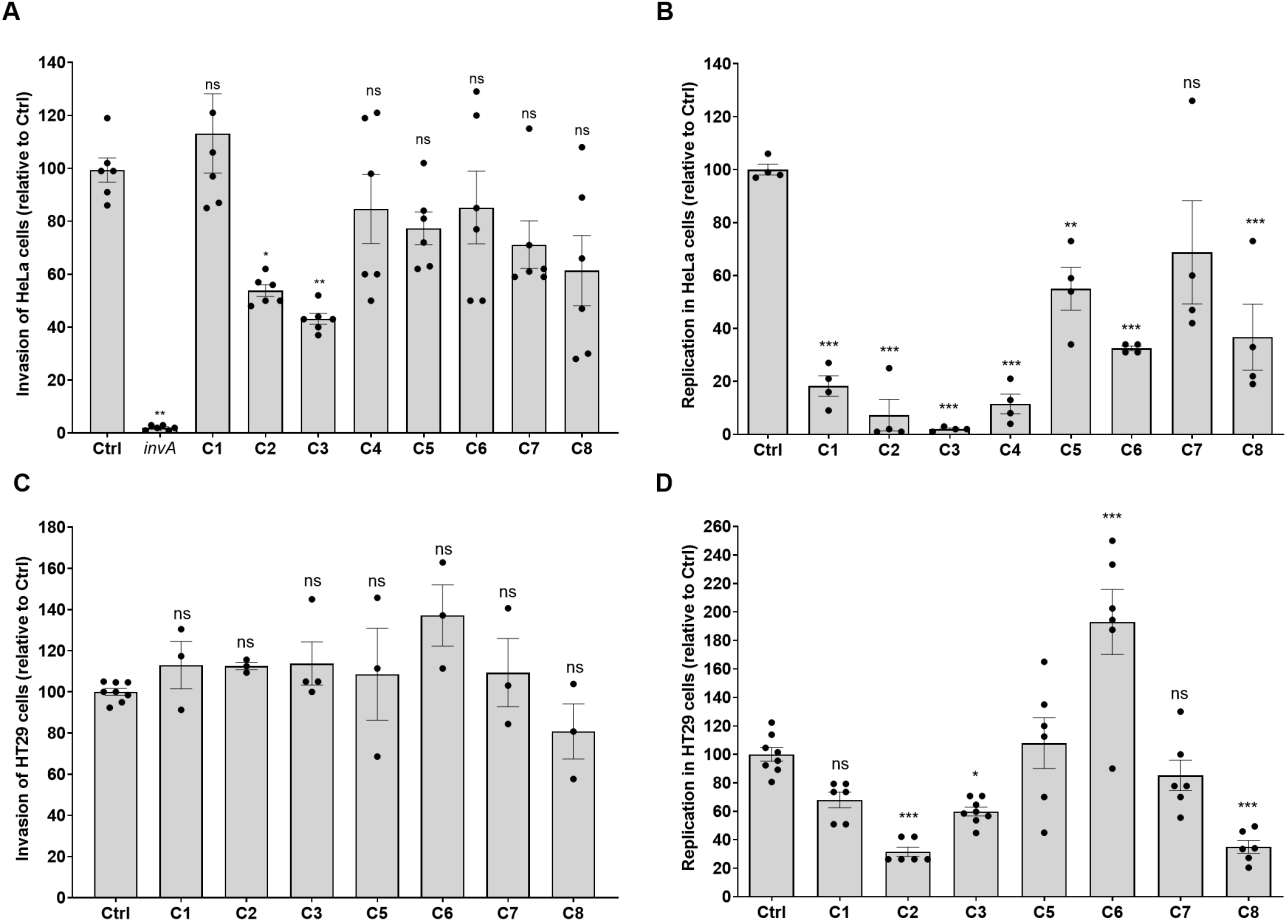
The effect of the identified compounds on host cell invasion and intracellular replication in epithelial cells. HeLa cells were seeded in a 24-well cell culture-treated microplate and incubated with fresh DMEM medium containing compounds C1 to C8 at a final concentration of 10 µM for 3 h prior to the infection. Cells were infected at an MOI of 50, incubated at 37°C under 5% CO_2_, and then subjected to the gentamicin protection assay as explained in the *Materials and methods* section to eliminate extracellular bacteria. At 2 hpi, cells were washed and lysed with lysis buffer. To calculate the number of invading bacteria at 2 hpi **(A)**, the cell lysates were serially diluted in PBS and plated onto LB-agar plates for CFU count. An *S*. Typhimurium *invA* null mutant strain impaired in invasion of non-phagocytic cells was included as a genetic control. To calculate the intracellular fold replication **(B)**, at 2 hpi, the medium of the infected cells was replaced with DMEM supplemented 10 µM of each compound and 10 µg/mL of gentamicin and incubated at 37°C in a humidified 5% CO_2_ incubator. At 24 h post-infection, the cells were washed and lysed as above. Intracellular replication was determined by the ratio between the number of intracellular cells counted at 24 hpi and their number at 2 hpi. The percentage of invasion and replication relative to HeLa cells infected with *S*. Typhimurium in the absence of the compounds (Ctrl) is shown. HT29-MTX-E12 cells were incubated with the indicated inhibitors for 3 h and infected with a mid-logarithmic culture of *S.* Typhimurium SL1344 at an MOI of 30. Infected cells were subjected to the gentamicin protection assay. **(C)** At 2 hpi, *Salmonella* invasion into HT29-MTX-E12 cells was determined as in **(A)**. **(D)** At 24 h after infection, cells were washed and lysed with lysis buffer. Serial dilutions were plated on LB agar plates for CFU counting, and intracellular replication was determined as in **(B)**. The charts show the mean and standard error of the mean (SEM) of at least three biological repeats. One-way ANOVA was used to determine statistical significance. *, P-value <0.05; **, P-value <0.01; ***, P-value < 0.001; ns, not statistically significant.

Given that the selected compounds were confirmed as non-cytotoxic with no culture growth inhibition, the reduced infection of host cells by *S.* Typhimurium in the presence of the compounds could be the result of their effect on the ability of *Salmonella* to invade host cells, replicate intracellularly, or both. To distinguish between these options, we next used a quantitative gentamicin protection assay (GPA) and determined directly, by intracellular CFUs counting, the ability of *S*. Typhimurium to enter and replicate within HeLa cells in the presence of these compounds. *Salmonella* invasion was assessed at 2 hpi, and its ability to replicate within these cells was evaluated at 24 hpi, in the presence and absence of the eight compounds at 10 µM concentration. As a control for *Salmonella* invasion, we used a *S*. Typhimurium mutant strain that lacks the *invA* gene (Δ*invA*), a structural gene of the T3SS-1, required for *Salmonella* invasion into non-phagocytic cells (Galan and Curtiss, 1991). As shown in **Figure 3A**, compounds C2 (PCM-0086166; penbutolol) and C3 (PCM-0004846) moderately inhibited *Salmonella* invasion into HeLa cells in a statistically significant manner. In agreement with this phenotype, both compounds C2 and C3 were also found to moderately reduce by 3- to 4-fold the transcription of the gene *invA*, but not the expression of SPI-4 genes involved in *Salmonella* adhesion to the epithelial cell surface (Bode et al., 1980) (**Supplementary Figure 2**). Other compounds were not found to affect SPI-1 gene expression in *S.* Typhimurium (data not shown). These results suggested that the impaired *Salmonella* invasion into epithelial cells might be mediated by downregulation of T3SS-1 genes conferred by compounds C2 and C3; however, a broader transcriptional analysis is required to better characterize the effect of these compounds on *Salmonella* gene expression.

Besides the reduced invasion in the presence of compounds C2 and C3, all of the eight compounds with the exception of compound C7 (PCM-0001349) demonstrated significant inhibition of *S.* Typhimurium replication within HeLa cells. Notably, although the inhibition of compound C7 on *Salmonella* replication did not reach statistical significance, the mean intracellular replication value was low by more than 30% relative to the *Salmonella* replication in the absence of the compounds (Ctrl; **Figure 3B**).

To verify these results in another epithelial cell line, we further tested the compounds (deprived of C4 that was temporarily unavailable for purchase), using a human colon-derived epithelial cell line, HT29-MTX-E12. While none of the inhibitors affected the invasion of *S.* Typhimurium into HT29-MTX-E12 cells (**Figure 3C**), compounds C2, C3, and C8 significantly reduced intracellular replication, while compound C6 unexpectedly increased *S.* Typhimurium replication within HT29-MTX-E12 cells (**Figure 3D**). We concluded from these experiments that at least three compounds (C2, C3, and C8) significantly impair the intracellular replication of *Salmonella* in different epithelial cell lines.

### Identified hits inhibit *Salmonella*’s intracellular survival in macrophages

**Figure 4:**
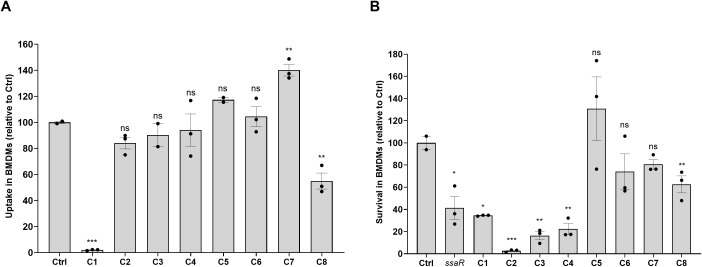
The effect of the identified compounds on *Salmonella* uptake by and intracellular survival in bone marrow-derived macrophages (BMDMs). BMDMs were isolated from the femur leg bone of SWISS female mice and seeded in a 24-well plate. BMDMs were incubated in fresh medium supplemented with 10 µM of the compounds for 3 h at 37 °C under 5% CO_2_ atmosphere prior to the infection. Macrophages were infected at an MOI of 10 with cultures of *S*. Typhimurium SL1344 and its Δ*ssaR* isogenic mutant that is impaired in intracellular replication as a negative control. The number of intracellular *Salmonella* was determined by the gentamicin protection assay, at 2 and 24 hpi. The number of CFUs in each well was quantified by plating serial dilutions of cell lysates on selective LB-agar plates. **(A)** *Salmonella* uptake was calculated as the number of intracellular bacteria recovered at 2 hpi, divided by the infecting inoculum (CFUs). **(B)** *Salmonella* survival was calculated as the number of intracellular bacteria recovered at 24 hpi, divided by intracellular bacteria recovered at 2 hpi. One-way ANOVA was used to determine statistical significance. *, P-value <0.05; **, P-value <0.01; ***, P-value < 0.001; ns, not statistically significant.

To test whether the inhibition of intracellular replication by these compounds is specific to epithelial cells only or confers replication inhibition in other cell types as well, we cultivated primary BMDMs from SWISS mice and infected them with *S*. Typhimurium. For that purpose, BMDMs were incubated for 3 h with 10 µM of compounds and infected at an MOI of 10. *Salmonella* uptake (**Figure 4A**) was calculated as the number of intracellular *Salmonella* CFUs recovered at 2 hpi, divided by the infecting inoculum. Intracellular survival (**Figure 4B**) was calculated as the number of intracellular bacteria recovered at 24 hpi, divided by the number of intracellular bacteria at 2 hpi. As a control for intramacrophage survival, we included its isogenic *ssaR* mutant strain (Δ*ssaR*) that is impaired in intracellular replication within phagocytic cells (Brumell et al., 2001). These analyses indicated that compounds C1 (PCM-0094889; saracatinib) and C8 (PCM-0095564; indacaterol maleate) can inhibit *Salmonella* uptake by BMDMs and that at least four compounds (C1, PCM-0094889, saracatinib; C2, PCM-0086166, penbutolol sulfate; C3, PCM-0004846; and C4, PCM-0103431) can significantly impair the intracellular replication of *S*. Typhimurium in BMDMs.

Overall, we concluded from the cell line infection experiments that the effect of these compounds on *Salmonella* interaction with host cells is not identical and can be mediated by inhibiting host cell entry, intracellular replication, or both and may vary between cell types. Nonetheless, five out of the eight tested molecules (C1, C2, C3, C4, and C8) inhibited intracellular replication of *Salmonella* within both HeLa and BMDMs.

### Most of the identified compounds also inhibit the intracellular growth of *Listeria monocytogenes*

**Figure 5:**
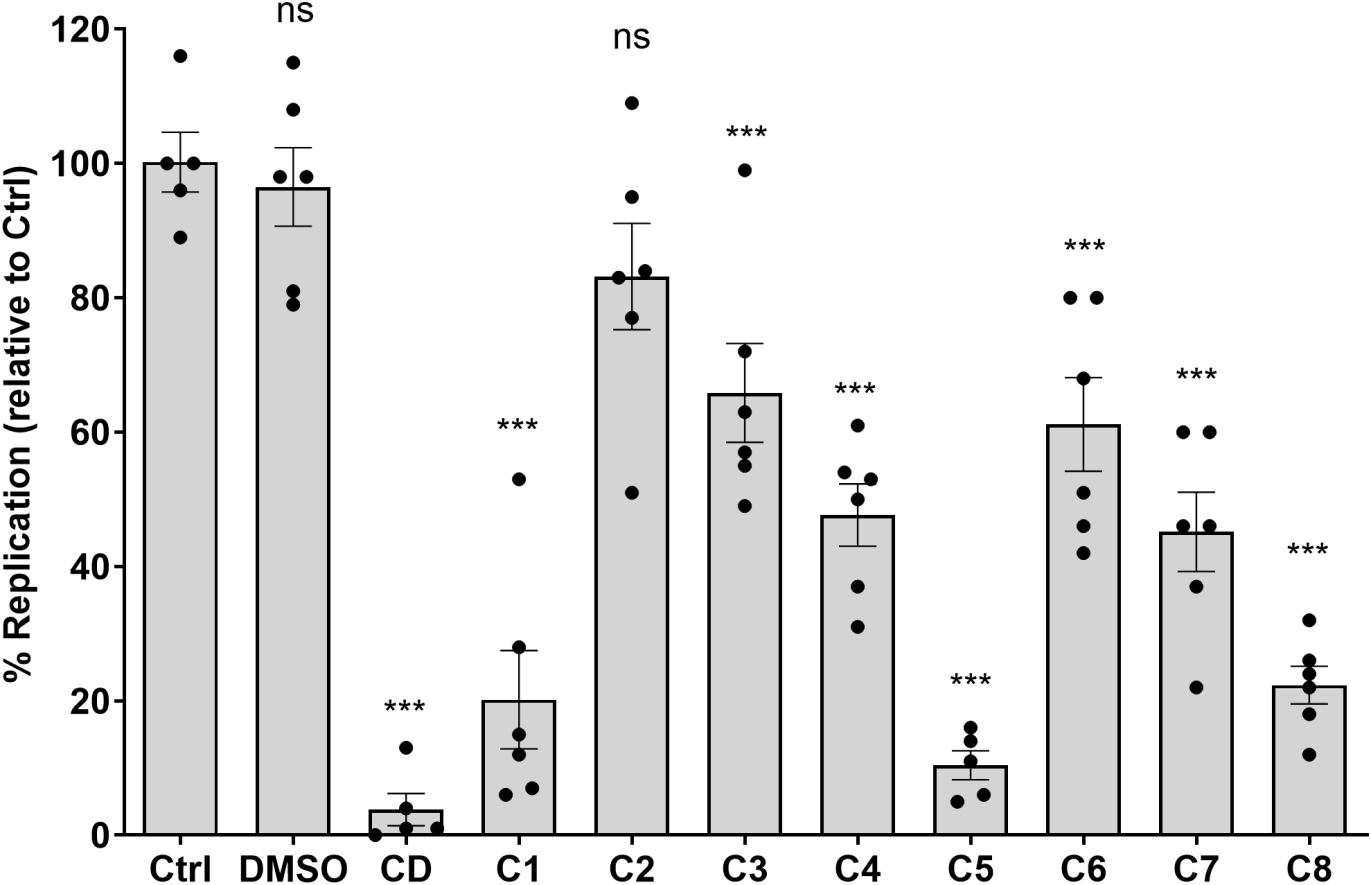
The identified compounds inhibit the intracellular growth of *Listeria monocytogenes*. HeLa cells were seeded in a 24-well tissue culture-treated plate and incubated for 3 h with fresh DMEM supplemented with C1 to C8 compounds at a final concentration of 10 µM. Overnight cultures of *L. monocytogenes* that were grown statically at 30 °C were washed, resuspended in PBS, and used to infect HeLa cells at an MOI of 50. One hour post-infection, gentamicin was added to each well at a final concentration of 50 µg/mL. At 6 hpi, the cells were washed with PBS and lysed as explained in the *Materials and methods* section. The cell lysates were then diluted in PBS and plated on LB plates for CFU counts. Intracellular *Listeria* infection was calculated as the number of intracellular bacteria recovered at 6 hpi, divided by the infecting inoculum (CFUs). *Listeria* infection in the presence of the compounds is shown relative to the number of intracellular bacteria that were recovered from cells that were not treated with the compounds (Ctrl). *Listeria monocytogenes* infections in the presence of 0.1% DMSO and CD were used as negative and positive controls, respectively. The charts show the mean and standard error of the mean (SEM) of at least five biological repeats. One-way ANOVA was used to determine statistical significance. ***, P-value < 0.001; ns, not statistically significant.

Inhibition of intracellular *Salmonella* growth by these compounds inspired us to test their activity against other intracellular pathogens. For that purpose, we studied the intracellular replication of the Gram-positive facultative intracellular bacterium, *L. monocytogenes*. HeLa cells that were incubated in DMEM (+10% FCS) supplemented with 10 µM of the eight compounds were infected with *L. monocytogenes* at an MOI of 1:50. One hour post-infection, gentamicin was added to each well at a final concentration of 50 µg/mL, and at 6 hpi, the cells were washed and lysed with double-distilled water. The quantity of intracellular *Listeria* was calculated as the number of intracellular CFUs recovered at 6 hpi, divided by the infecting inoculum. The CFU of intracellular *Listeria* recovered from treated cells was compared to the number of intracellular bacteria that were obtained from untreated cells (Ctrl). As a positive control, we again used CD that blocks *Listeria* invasion into HeLa cells (Mounier et al., 1990). As shown in **Figure 5**, all compounds besides compound C2 (PCM-0086166; penbutolol) significantly inhibited the intracellular growth of *L. monocytogenes* in HeLa cells. Noticeably, compounds C1, C5, and C8 presented the strongest inhibition, resulting in a mean inhibition of more than 75%, relative to Ctrl. We concluded from these results that the ability of the tested compounds to inhibit intracellular growth of bacterial pathogens is not specific to *Salmonella* only and that they can be similarly effective against other bacterial species, including Gram-positive pathogens.

### Structure–activity relationship studies for compound C4

**Figure 6:**
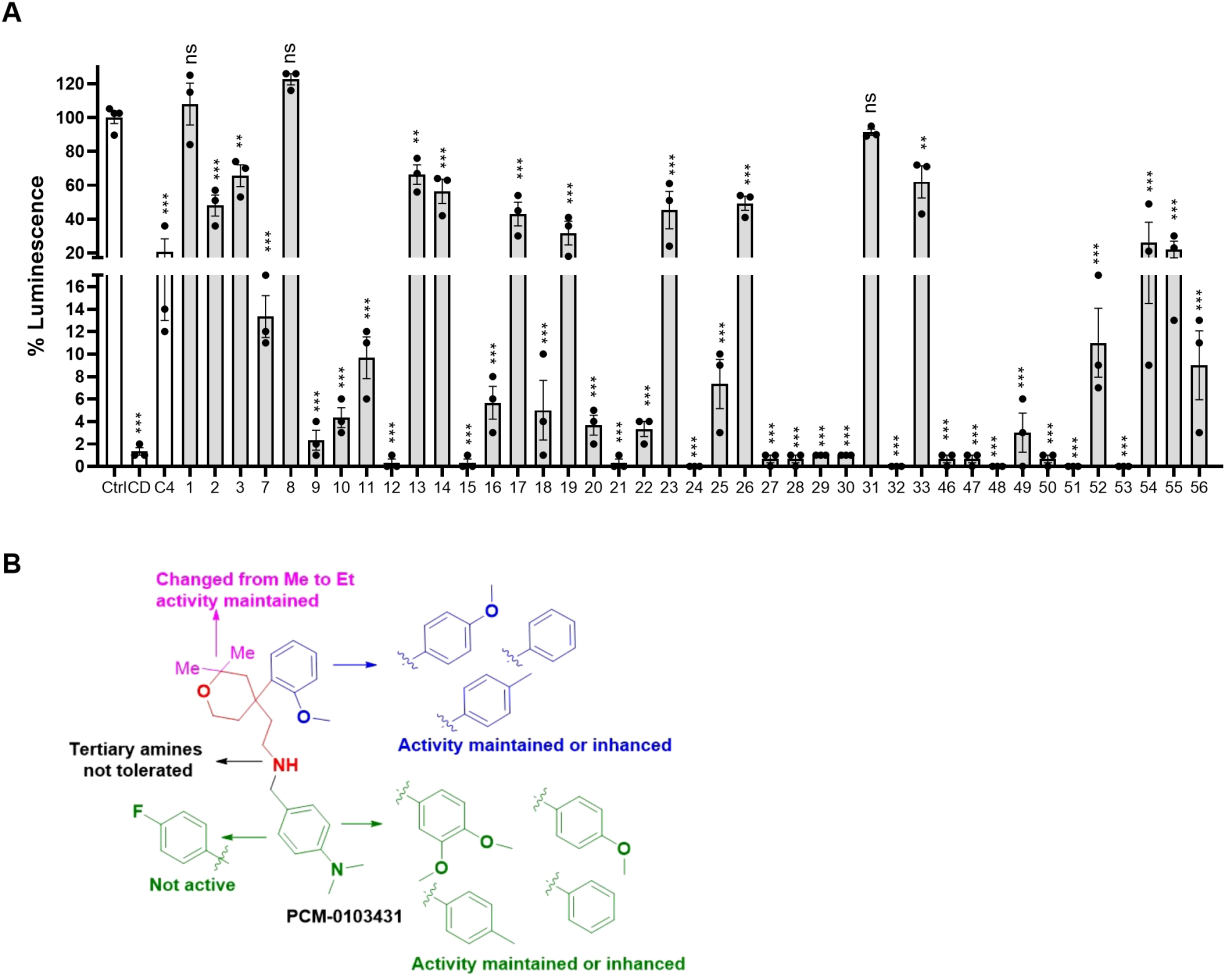
Structure–activity relationship (SAR) studies of compound C4. **(A)** HeLa cells were seeded in a 96-well, cell culture-treated, microplate and incubated at 37°C in 5% CO_2_ humidified incubator for 4 h in the presence of 10 µM of compound C4 (PCM-0103431) and its chemical analogs (shown as gray bars). As a positive control, 2 µM of cytochalasin D (CD) was also included. Cells were infected with subcultures of *S*. Typhimurium expressing the reporter system P*ssek3::lux* at an MOI of 50. One hpi, gentamicin was added to a final concentration of 20 µg/mL, and the infected cells were incubated overnight, until bioluminescence was read. Host cell infection is shown as the intracellular luminescence relative to its value in HeLa cells infected with *S.* Typhimurium in the presence of 0.1% DMSO (Ctrl). The charts show the mean and SEM of three biological repeats. One-way ANOVA was used to determine statistical significance. **(B)** SAR diagram of compound 4 (PCM-0103431) of various modifications made to the pyran scaffold. In green, the aniline moiety was substituted with electron-donating and neutral groups. In red, changes to the amine were not tolerated; a secondary amine was crucial for activity. Magenta: methyl groups could be replaced with ethyl, maintaining activity. Blue: p-methoxypheny, tolyl, and benzyl groups maintained their activity. **, P-value <0.01; ***, P-value < 0.001; ns, not statistically significant.

In order to identify functional groups of compounds that contribute to the inhibition of *Salmonella* infection and to potentially improve their activity, we applied a SAR approach, as a proof of concept for compound C4 (PCM-0103431), which inhibited the intracellular growth of *Salmonella* and *Listeria* in HeLa cells and *Salmonella* survival in macrophages. Forty-one commercially available related analogs of compound C4 (**Supplementary Table 5**) were obtained and tested for their ability to inhibit *S*. Typhimurium infection of human epithelial cells. These chemical analogs were selected according to their similarity to the pyran ring of compound C4. As before, *S*. Typhimurium carrying P*sseK3::lux* was used to infect HeLa cells, in the presence of 10 µM of each molecule, and the infection efficiency was determined by quantifying their intracellular bioluminescence at 16 hpi. Interestingly, 15 analogs of compound C4 demonstrated lower intracellular luminescence than the original compound (**Figure 6A**), indicating that the original inhibitory activity of compound C4 can be further improved by chemical modification.

Based on the resulting biological activity, we were able to identify essential, non-essential, and replaceable functional groups in compound C4 (**Figure 6B**). This analysis showed that the pyran scaffold (shown in red) is essential and that the substitution of the anisole group (shown in blue) with electron donating neutral or withdrawing such as fluorine groups either enhanced or maintained the inhibition activity of the molecule. This approach also demonstrated that the presence of the secondary amine was important for its infection inhibition activity, as analogs with a tertiary amine were less potent. Finally, we found that the aniline group (shown in green) could be replaced with electron-donating or neutral groups without affecting its biological activity.

Importantly, analogs 21, 30, 46, and 50 of compound 4 that showed improved activity were further tested for their cytotoxicity against host cells and assayed for their antibacterial activity against extracellular *Salmonella*. These analyses showed that at 10 µM, the tested analogs were non-cytotoxic and not antibacterial (**Supplementary Figure 3**), suggesting that they may be developed as improved inhibitors against *Salmonella* infection.

## Discussion

While the development of new classes of antibiotics is one pivot in fighting antibiotic resistance, HDTs that target host-mediated functions necessary for bacterial infection, replication, and pathogenesis have the potential to be an alternative or adjunctive approach to combat bacterial infections (Varma et al., 2020). In contrast to antibiotics that directly act on the bacterial cell and therefore pose a strong selective pressure, HDTs enhance a potentially broad response against the bacteria, rendering host cells less permissive for bacterial growth, without granting a strong selective pressure and thereby limiting the development of drug resistance (Kilinc et al., 2021; Liu et al., 2022).

Latest advances in HDTs have provided promising, emerging approaches to reduce or eliminate intracellular bacterial infections, by targeting a variety of host factors that can restrict the replication and persistence of pathogens inside the cell (Kilinc et al., 2021; Liu et al., 2022). As such, host-directed approaches to combat bacterial infections have gained an impressive momentum in the recent decade (Zumla et al., 2016) and were reported to be effective against multiple pathogens including *M. tuberculosis* (Mahajan et al., 2025), *S.* Typhimurium (Van Den Biggelaar et al., 2024; Sahile et al., 2025), *Klebsiella pneumoniae* (Abhinand et al., 2025), *Acinetobacter baumannii* (Singothu et al., 2025), extraintestinal pathogenic *E. coli* (ExPEC) (Jiang et al., 2025), and *Streptococcus pneumoniae* (Sundaresh et al., 2021), as well as various viral infections (Kaufmann et al., 2018; Vinh, 2025). Nevertheless, the ability to identify suitable molecules that could be used for effective and safe HDT is largely limited by the initial size of the compound library and the screening protocol.

Here, we applied a cell-based HTS using a diverse collection of nearly 37,000 potentially pharmacologically active molecules to identify compounds that can inhibit host cell infection and intracellular proliferation of *S*. Typhimurium. One strength of this screen was the high number of the tested compounds, which exceeded by more than 10-fold, previously reported screens, performed to find host-targeted therapeutic candidates against *S.* Typhi (Hoang et al., 2022) and invasive non-typhoidal *Salmonella* (iNTS) (Tsai et al., 2023). Another point of novelty in this screen was the utilization of an intracellularly induced luminescence marker, cloned under the control of the T3SS-2 gene, *sseK3*. Using this reporting system as a readout for the intracellular *S.* Typhimurium load contributed to the specificity and sensitivity of the screen and facilitated the identification of eight compounds that inhibit the intracellular replication and/or the invasion of *Salmonella* within epithelial cells, in a dose-dependent manner. Additionally, five of these eight molecules also reduced *Salmonella* intracellular replication in BMDMs. In contrast, extracellular cell-independent bacterial growth was unaffected by these compounds, indicating that they do not act directly as antibacterial drugs, and therefore, the chances of developing bacterial resistance in the absence of a direct negative selection are low.

Importantly, seven out of the eight identified compounds were also found to inhibit the intracellular growth of the Gram-positive bacteria *L. monocytogenes*, and one compound (C1/PCM-0094889) was recently shown to inhibit the intramacrophage survival of *M. tuberculosis* (Chandra et al., 2016). Another compound with a previously suggested anti-*M. tuberculosis* activity is compound C6 (conivaptan), which was identified using a virtual screening as a putative EPSP synthase inhibitor in *M. tuberculosis* (Dahmer et al., 2023). EPSP synthase is the enzyme responsible for the sixth step of the shikimate pathway (De Oliveira et al., 2020), essential in mycobacteria, but absent in humans and therefore was proposed as a potential target for the development of a new tuberculosis treatment (Dahmer et al., 2023).

Collectively, our new results together with previously published reports indicate that a subset of the identified compounds inhibit the intracellular growth of very different and phylogenetically distant bacteria at an effective concentration of 10 µM. This conclusion suggests that the identified compounds probably target host factors/processes, which are commonly required by intracellular pathogens to establish or maintain host infection.

Although the main effect of the identified compounds is likely directed toward host factors, two compounds (C2 and C3) were also found to downregulate the expression of a structural gene of the *Salmonella* T3SS-1 apparatus (*invA*). These results suggest an interesting possibility pointing to a dual activity of these compounds, which may be active against both host and pathogen pathways. Previously, a similar approach of HTS of a small molecule library successfully identified compounds that inhibit the T3SS of the bacterial pathogen *Yersinia pestis* (Pan et al., 2007). Identification of new molecules that can target both host and pathogen components is intriguing and may lead to the development of highly effective novel therapeutics.

Despite the strengths of our findings, one of the main limitations of this study is that the exact mechanisms through which these molecules restrict host cell infection are unclear and are not straightforward to identify. These compounds may affect different cellular pathways that augment the antimicrobial response of the cells including reactive oxygen species generation, autophagy, phagolysosome maturation, antimicrobial peptide production, enhancing Toll-like receptor signaling, cell metabolism, and more. Recently, a genome-scale CRISPR knockout library screen in THP-1 human macrophages was employed to reveal host genes required for *S.* Typhimurium infection (Yeung et al., 2019). The screen identified 186 host genes involved in various cellular pathways, including actin dynamics, glycosaminoglycan and cholesterol metabolism, receptor signaling, lipid raft formation, and calcium transport, highlighting the diverse mechanisms involved in cell infection and their potential as targets for host-directed therapy.

Although we cannot exclude the direct effect on the pathogen itself under intracellular conditions, for five out of the eight identified infection inhibitors, the biological/pharmaceutical function is known and may point toward a potential mechanism that can explain their effect on host cells in the context of infection. Compound C1 (saracatinib or AZD0530) is a tyrosine kinase inhibitor that primarily targets Src/Abl family kinases (Hennequin et al., 2006). Previous studies have shown the involvement of c-Src tyrosine kinase in *Salmonella* Enteritidis Rck protein-mediated invasion (Wiedemann et al., 2012) and that cellular Abelson tyrosine kinase facilitates *S*. Typhimurium entry into epithelial cells (Ly and Casanova, 2009). AZD0530 was recently shown to attenuate the survival of *M. tuberculosis* in THP-1 macrophages and to reduce the bacterial burden and disease pathology in a guinea pig infection model (Chandra et al., 2016). Moreover, AZD0530 was reported to block signaling through the Src/phosphatidylinositol 3-kinase (PI3K)/AKT axis, which affects many downstream eukaryotic signaling pathways (Datta et al., 1996; Haynes et al., 2003), including autophagy (Kroemer et al., 2010), that serves as a potent mechanism to eliminate intracellular pathogens (Riebisch et al., 2021).

Another identified compound with a putative tyrosine kinase inhibition activity is compound C5 (PCM-0095494). This molecule is an Arry-380 analog that is known as a powerful HER2 (human epidermal growth factor receptor 2) tyrosine kinase inhibitor, which selectively binds to and inhibits the phosphorylation of HER2 (Martin and Lopez-Tarruella, 2016; Moulder et al., 2017). Because many bacterial pathogens including *Pseudomonas aeruginosa*, *Shigella flexneri*, and *Helicobacter pylori* exploit host Abl family tyrosine kinases for their pathogenicity (Burton et al., 2003; Tammer et al., 2007; Pielage et al., 2008; Ly and Casanova, 2009), inhibitors of this class could serve as an effective, wide-range approach to control infection by different intracellular pathogens. This strategy has been successfully demonstrated in previous studies, and several PI3K inhibitors, which are already FDA-approved for cancer therapy, were shown to decrease intracellular survival within macrophages of *S*. Typhimurium, *K. pneumoniae*, and *M. tuberculosis*. Moreover, PI3K inhibitors were also shown to reduce bacterial invasion and survival within epithelial cells of *S*. Typhimurium, *H. pylori*, and *L. monocytogenes* and the penetration of the blood–brain barrier by *E. coli* leading to meningitis (Fleeman, 2023).

Compound C2 (penbutolol) is a beta-adrenergic blocking agent and compound C8 (indacaterol maleate) is an ultra-long-acting beta-2 adrenergic agonist. Several studies have examined how β-adrenoceptor blockers influence bacterial quorum-sensing (QS) systems (Almalki et al., 2022; Thabit et al., 2022). These reports demonstrate that different β-blockers reduce QS-regulated biofilm formation in *P. aeruginosa* and *S.* Typhimurium and downregulate QS-associated genes in *P. aeruginosa* as well as genes encoding bacterial adrenergic sensor kinases in *S.* Typhimurium, although at relatively high concentrations of 500 µg/mL (Almalki et al., 2022). A recent study reported that the β-adrenoreceptor blocker atenolol was able to reduce *S.* Typhimurium biofilm formation, invasion into HeLa cells, and intracellular replication inside macrophages. Moreover, Thabit and colleagues also showed that this β-blocker significantly downregulated T3SS-2 genes, the quorum sensing receptor, *sdiA*, and norepinephrine membrane sensors *qseC* and *qseE*, and that atenolol treatment protected mice from *S*. Typhimurium infection (Thabit et al., 2022). Therefore, these data may infer possible directions for the effect of compounds C1, C2, C5, and C8 on the interaction of *Salmonella* with host cells; however, further follow-up studies are still required to confirm these putative mechanisms.

In summary, this study provides a methodological platform and a cell-based high-throughput screening approach, combined with an intracellular-induced reporter to identify host-directed therapy candidates against intracellular pathogens and demonstrates the efficacy of the SAR approach to optimize these hits. Nevertheless, further study is warranted to recognize their specific host targets and their particular mechanisms of actions. So far, most HDTs were proposed for use as adjuvant treatments, and their antimicrobial properties are generally undercharacterized. Future research that will focus on elucidating their host targets, optimizing their chemical structure, and evaluating their efficacy in clinical studies will contribute significantly to the ongoing battle against the antibiotic resistance pandemic and the global burden of infectious diseases caused by intracellular pathogens.

## Data Availability

All relevant data is contained within the article: The original contributions presented in the study are included in the article/**Supplementary Material**, further inquiries can be directed to the corresponding author.

## Supplementary material

Supplementary Figure 1 Dose-response relationship and cytotoxicity assay of the eight compounds. **(A)** The ability of the tested compounds (C1 to C8) to inhibit HeLa cells infection by *S.* Typhimurium carrying the P*sseK3::lux* reporter was studied under increasing drug concentrations of 0.25, 1, 3.25, 10, and 30 µM. *Salmonella* infection was conducted by the gentamicin protection assay as explained in the Materials and Methods and luminescence was read 16 h post infection. Luminescence is shown in % relative to the luminescence of cells that were infected with the reporter strain in the absence of compounds. Each point indicates the mean of two independent infections in one experiment. (**B**) Cytotoxicity of the compounds was tested by the CellTiter-Glo (GTG) Luminescent Cell Viability Assay in the presence of the compounds at a final concentration of 0.3, 1, 3, 10, and 30 µM. Compounds were incubated with HeLa (brown) or HB2 (blue) epithelial cells for 5 h at 37°C under 5% CO2 atmosphere before cell Viability was evaluated.

Supplementary Figure 2 Compounds C2 and C3 downregulate the expression of a T3SS-1 gene in *Salmonella*. *S*. Typhimurium SL1344 were subcultured 1:100 into fresh LB medium in the presence of 10 µM compounds C2 (PCM-0086166) and C3 (PCM-0004846). The cultures were grown for 3 h to the late logarithmic phase (OD_600_ ~ 1.0). Relative quantification of target transcripts was determined using the comparative threshold cycle (CT) method and normalized to the housekeeping gene *rpoD*. Fold change in gene expression is shown for *sipB, invA, siiA*, and *siiC*. The bars show the mean of three biological repeats, while the SEM is indicated by error bars.

Supplementary Figure 3 The cytotoxicity and antibacterial activity of compound C4 analogs. The cytotoxicity of the chemical analogs 21, 30, 46 and 50 of compound C4 was determined using the CellTiter-Glo (GTG) Luminescent Cell Viability Assay in the presence of 0.3, 1, 3, 10, and 30 µM compounds in HeLa (brown) or HB2 (blue) epithelial cells for 5 h at 37°C under 5% CO2 atmosphere (Left panels). The antibacterial activity of the analogs was tested by comparing the growth curves of *S*. Typhimurium that were subcultured 1:100 in LB without compounds (STM; orange line), in the presence of 0.1% DMSO (blue line), and 10 µM compounds (black line) over 26 h at 37°C with shaking (Right panels).

Supplementary Table 1 Bacterial strains and plasmids used in this study. SGSC, *Salmonella* genetic Stock Center at the University of Calgary.

Supplementary Table 2 Primers used in this study

Supplementary Table 3 List of compounds that were examined in the HTS. 36,855 small molecules obtained from different suppliers were purchased and assembled as a comprehensive library of small bioactive molecules. Molecule name, its chemical structure, supplier, and catalog number, are given in the table.

Supplementary Table 4 Compounds that inhibited bioluminescence production in *Salmonella*

Supplementary Table 5 A list of forty-one chemical derivatives of compound C4 analogs used for SAR studies.
