# Supplementary figures and images for: An exhaustive cell-based screen coupled with an intracellular-induced lux-based reporter identified bioactive molecules that inhibit host cell infection by intracellular pathogens
Source: Front Cell Infect Microbiol. 2026 Mar 9;16:1770677. doi: 10.3389/fcimb.2026.1770677 (PMC13006506; doi:10.3389/fcimb.2026.1770677)

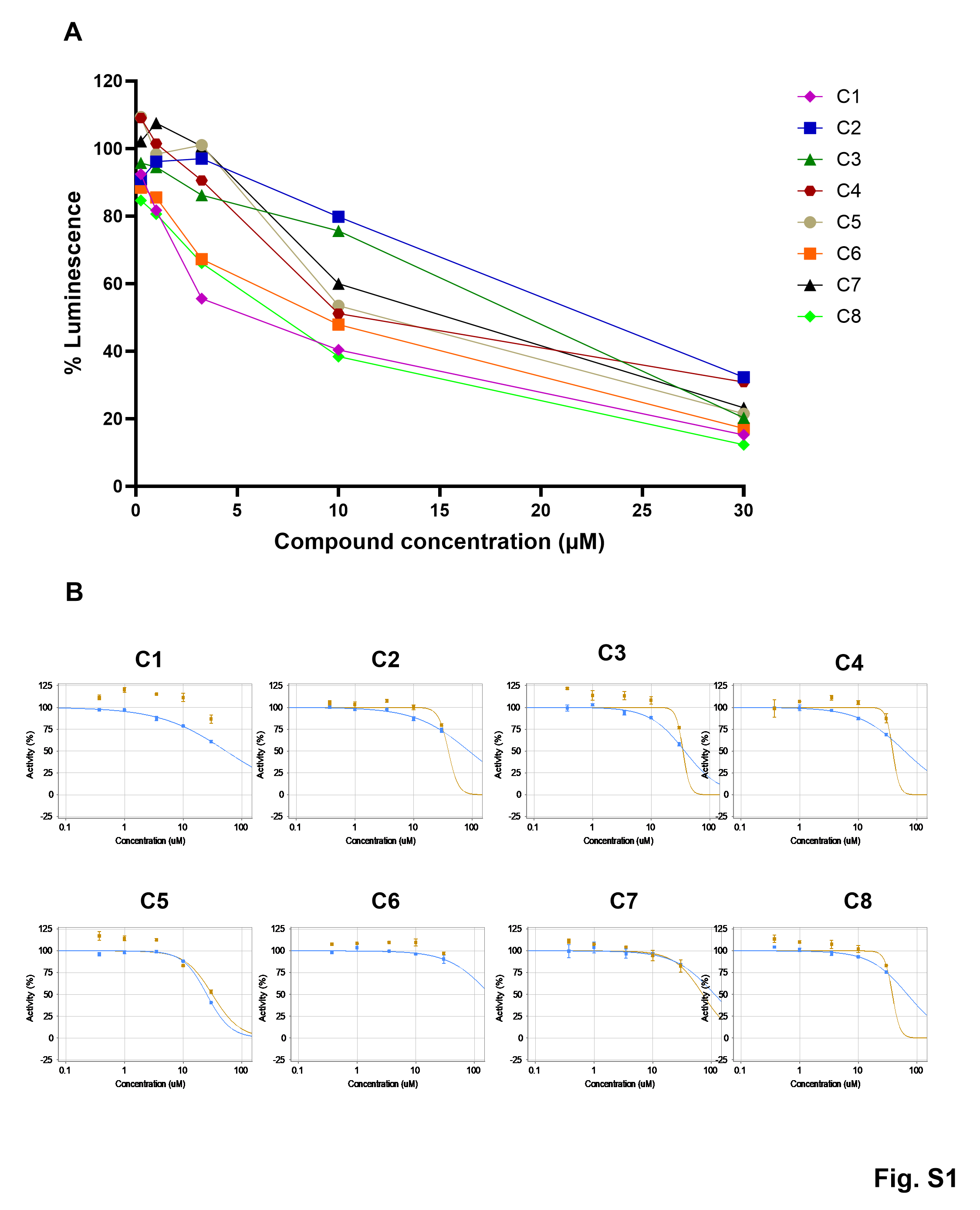

Supplement: Supplementary Figure 1 — Dose-response relationship and cytotoxicity assay of the eight compounds. (A) The ability of the tested compounds (C1 to C8) to inhibit HeLa cells infection by S. Typhimurium carrying the PsseK3::lux reporter was studied under increasing drug concentrations of 0.25, 1, 3.25, 10, and 30 µM. Salmonella infection was conducted by the gentamicin protection assay as explained in the Materials and Methods and luminescence was read 16 h post infection. Luminescence is shown in % relative to the luminescence of cells that were infected with the reporter strain in the absence of compounds. Each point indicates the mean of two independent infections in one experiment. (B) Cytotoxicity of the compounds was tested by the CellTiter-Glo (GTG) Luminescent Cell Viability Assay in the presence of the compounds at a final concentration of 0.3, 1, 3, 10, and 30 µM. Compounds were incubated with HeLa (brown) or HB2 (blue) epithelial cells for 5 h at 37°C under 5% CO2 atmosphere before cell Viability was evaluated. [file Image1.tif]

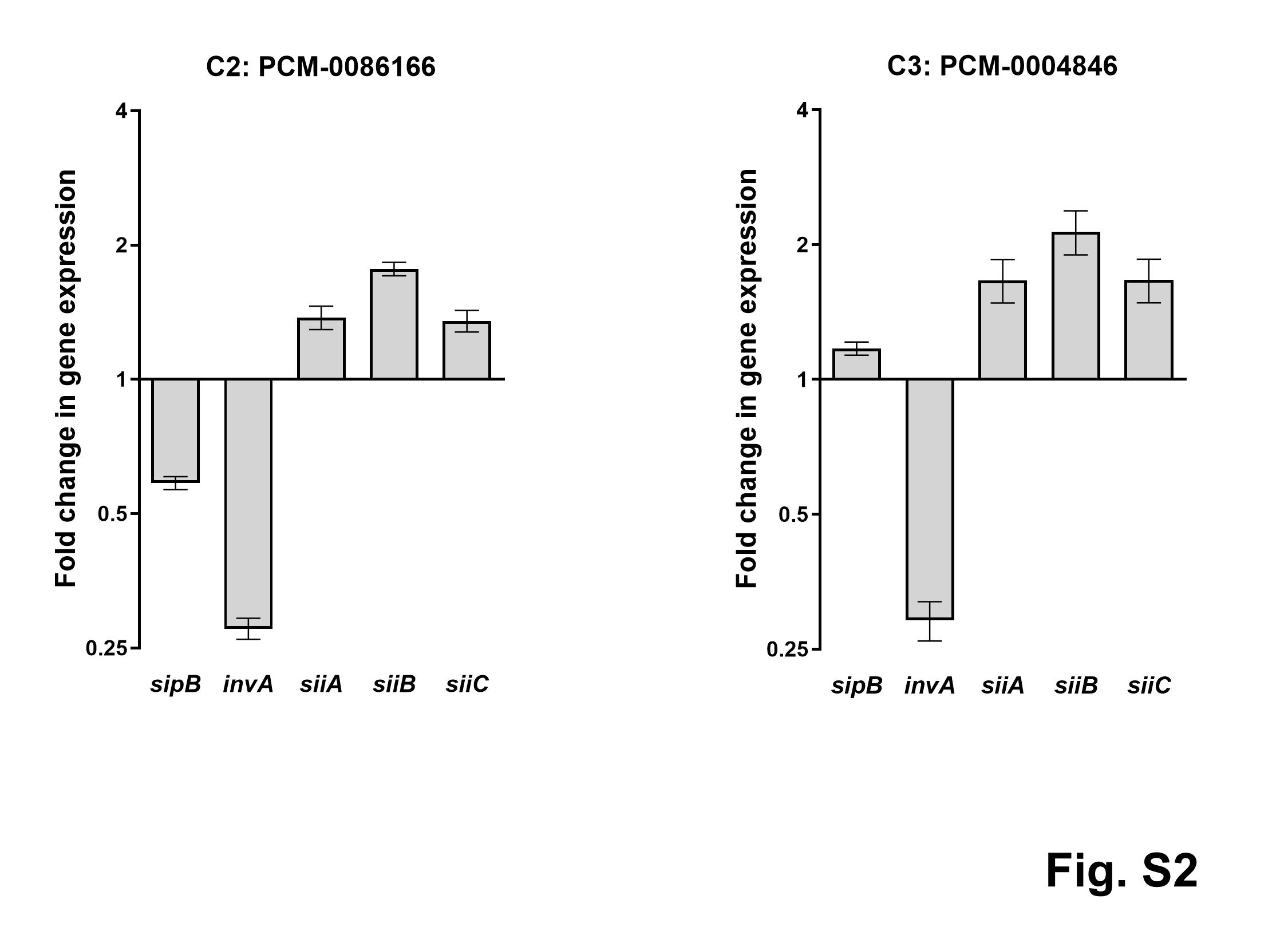

Supplement: Supplementary Figure 2 — Compounds C2 and C3 downregulate the expression of a T3SS-1 gene in Salmonella. S. Typhimurium SL1344 were subcultured 1:100 into fresh LB medium in the presence of 10 µM compounds C2 (PCM-0086166) and C3 (PCM-0004846). The cultures were grown for 3 h to the late logarithmic phase (OD600 ~ 1.0). Relative quantification of target transcripts was determined using the comparative threshold cycle (CT) method and normalized to the housekeeping gene rpoD. Fold change in gene expression is shown for sipB, invA, siiA, and siiC. The bars show the mean of three biological repeats, while the SEM is indicated by error bars. [file Image2.tif]

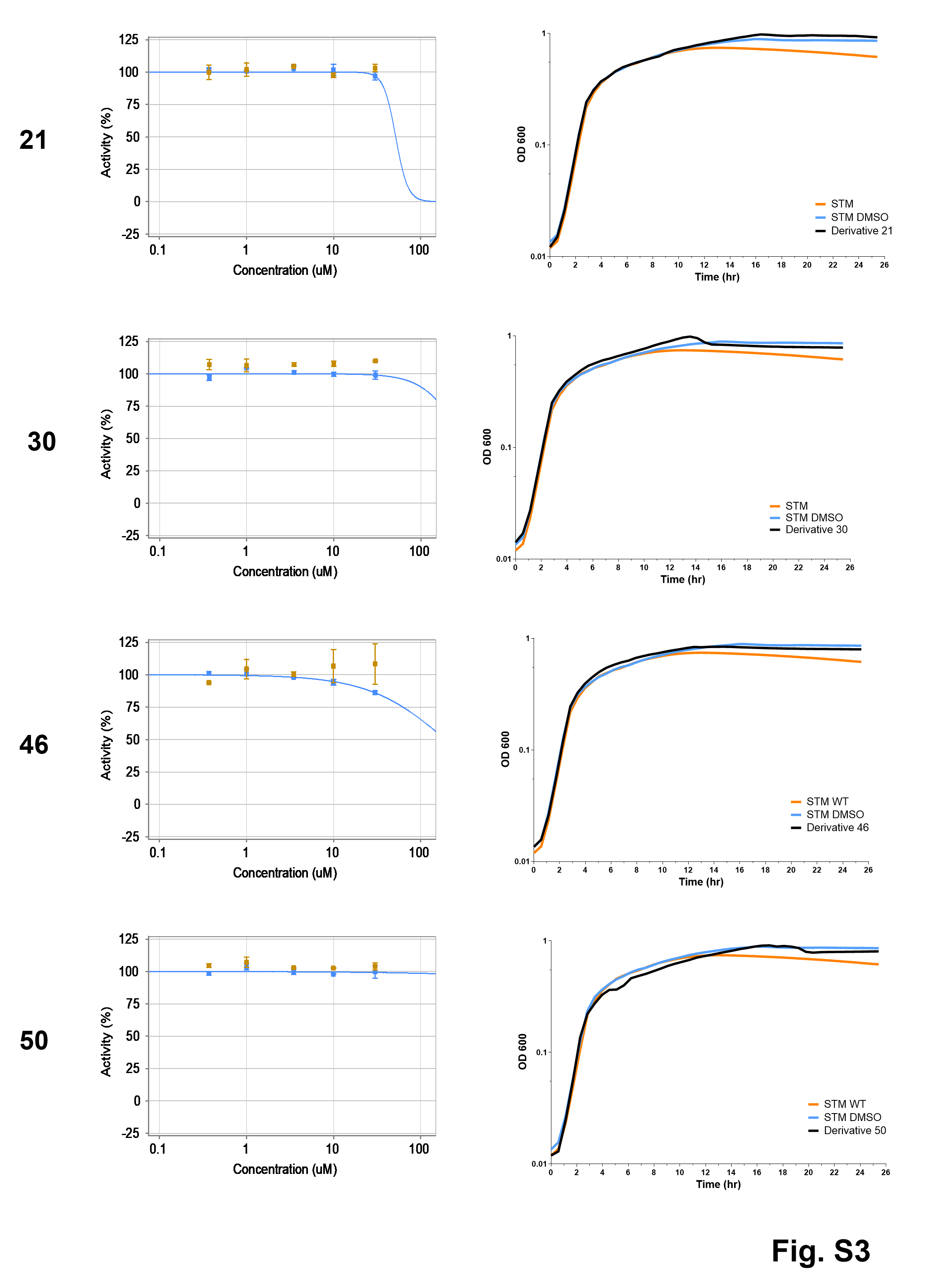

Supplement: Supplementary Figure 3 — The cytotoxicity and antibacterial activity of compound C4 analogs. The cytotoxicity of the chemical analogs 21, 30, 46 and 50 of compound C4 was determined using the CellTiter-Glo (GTG) Luminescent Cell Viability Assay in the presence of 0.3, 1, 3, 10, and 30 µM compounds in HeLa (brown) or HB2 (blue) epithelial cells for 5 h at 37°C under 5% CO2 atmosphere (Left panels). The antibacterial activity of the analogs was tested by comparing the growth curves of S. Typhimurium that were subcultured 1:100 in LB without compounds (STM; orange line), in the presence of 0.1% DMSO (blue line), and 10 µM compounds (black line) over 26 h at 37°C with shaking (Right panels). [file Image3.tif]
