# Supplementary material for: An exhaustive cell-based screen coupled with an intracellular-induced lux-based reporter identified bioactive molecules that inhibit host cell infection by intracellular pathogens
Source: Front Cell Infect Microbiol. 2026 Mar 9;16:1770677. doi: 10.3389/fcimb.2026.1770677 (PMC13006506; doi:10.3389/fcimb.2026.1770677)
Supplement: Supplementary Table 1 — Bacterial strains and plasmids used in this study. SGSC, Salmonella genetic Stock Center at the University of Calgary. [file Table1.docx]

**Table S1. Bacterial strains and plasmids used in this study**

| Strain or plasmid | Genotype and description | Reference or source |
| --- | --- | --- |
| *S.* Typhimurium SL1344 | wild type Sm^r^ *xyl hisG rpsL* | SGSC |
| *S.* Typhimurium *invA* | SL1344 Δ*invA* | (Galan and Curtiss, 1991) |
| *S.* Typhimurium *ssaR* | SL1344 Δ*ssaR* |  |
| *E. coli* DH5α | *recA1 endA1 lacZΔM15* | Gal-Mor Lab collection |
| *Listeria monocytogenes* EDG | wild type | Hensel lab collection |
| Plasmids |  |  |
| pCS26 | Kan^r^, low-copy number cloning vector for *luxCDABE* fusion | (Bjarnason et al., 2003) |
| pCS26::P*sseK3* | *S*. Typhimurium SL1344 *sseK3* regulatory region cloned into pCS26 | This study |
| pCS26::P*rpoD* | *S*. Typhimurium SL1344 *rpoD* regulatory region cloned into pCS26 | (Bjarnason et al., 2003) |

SGSC – *Salmonella* genetic Stock Center at the University of Calgary.

**REFERENCES**

Bjarnason, J., Southward, C.M., and Surette, M.G. (2003). Genomic profiling of iron-responsive genes in Salmonella enterica serovar typhimurium by high-throughput screening of a random promoter library. *J Bacteriol* 185**,** 4973-4982.

Galan, J.E., and Curtiss, R., 3rd (1991). Distribution of the invA, -B, -C, and -D genes of Salmonella typhimurium among other Salmonella serovars: invA mutants of Salmonella typhi are deficient for entry into mammalian cells. *Infect Immun* 59**,** 2901-2908.
