# Supplementary material for: An exhaustive cell-based screen coupled with an intracellular-induced lux-based reporter identified bioactive molecules that inhibit host cell infection by intracellular pathogens
Source: Front Cell Infect Microbiol. 2026 Mar 9;16:1770677. doi: 10.3389/fcimb.2026.1770677 (PMC13006506; doi:10.3389/fcimb.2026.1770677)
Supplement: Supplementary Table 2 — Primers used in this study [file Table2.docx]

**Table S2. Primers used in this study**

| **Primer name** | **Sequence (5' to 3')** | **Purpose** |
| --- | --- | --- |
| F XhoI sseK3 Pro’ | ccg**CTCGAG**AAAACATATCGTATAAGAAGTATAAAATAC | sseK3 promoter cloning into pCS26 |
| R BamHI sseK3 Pro | cgc**GGATCC**CAACCCTTACGCTATTAATATAAC |  |
| sipB_Fw | ACTGGAGTCTCGTCTGGCG | RT-PCR of *sipB* |
| sipB_Rev | GTCATAAACACTCTTGGCGGTA |  |
| invA_Fw | TCCGCTAATTTGATGGATCTCA | RT-PCR of *invA* |
| invA_Rev | ATCCGGAAAACGACCTTCAATC |  |
| siiA_Fw_SL1344 | CGACTGGGATATGAACGGGG | RT-PCR of *siiA* |
| siiA_Rev_SL1344 | CGTTGTACTTGATGCTGCGG |  |
| siiB_Fw_SL1344 | TGACAATGTGTCTACCGCCC | RT-PCR of *siiB* |
| siiB_Rev_SL1344 | TAGACAGACCCGCAAACGTC |  |
| siiC_Fw_SL1344 | GGCAAGTTACTCGCAGGCTA | RT-PCR of *siiC* |
| siiC_Rev_SL1344 | CTCTTTTGCAGCGGCGTATC |  |
