# Supplementary material for: An exhaustive cell-based screen coupled with an intracellular-induced lux-based reporter identified bioactive molecules that inhibit host cell infection by intracellular pathogens
Source: Front Cell Infect Microbiol. 2026 Mar 9;16:1770677. doi: 10.3389/fcimb.2026.1770677 (PMC13006506; doi:10.3389/fcimb.2026.1770677)
Supplement: Supplementary Table 4 — Compounds that inhibited bioluminescence production in Salmonella [file Table4.docx]

**Table S4. Compounds that inhibited bioluminescence production in *Salmonella***

|  | **Molecule Name** | **Structure** | **Supplier** | **Library** | **Luminescence inhibition (in %) at 10 µM relative to mock** |
| --- | --- | --- | --- | --- | --- |
| 1 | PCM-0003371 | 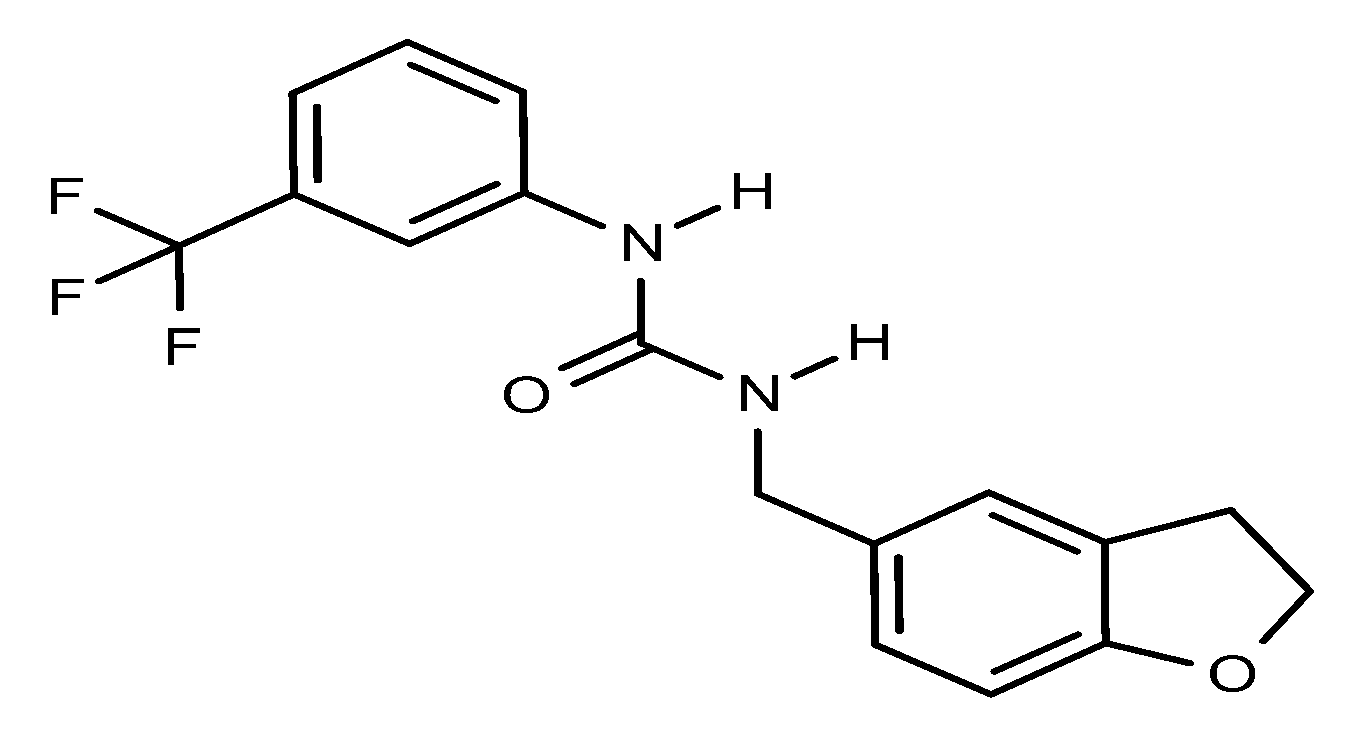 | MayBridge | HitFinder | 22 |
| 2 | PCM-0104441 | 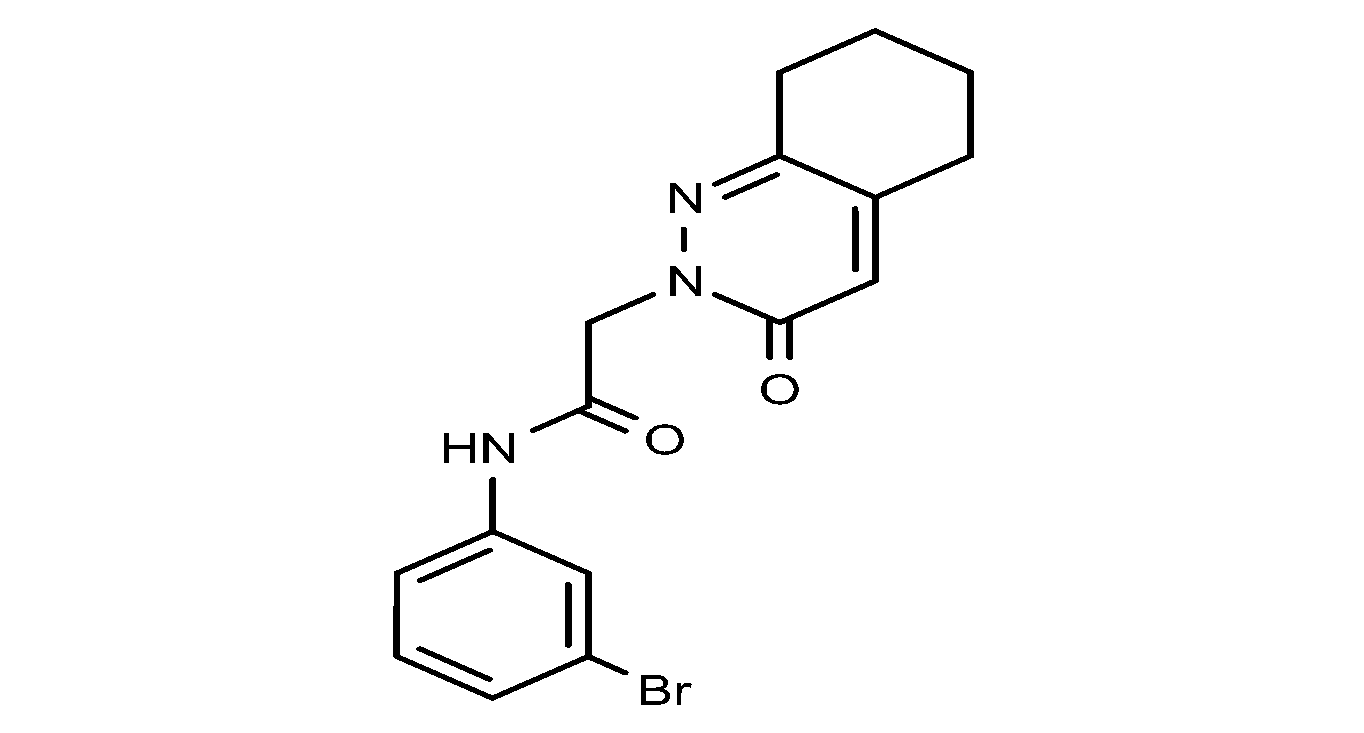 | ChemDiv | 100K | 12 |
| 3 | PCM-0104165 | 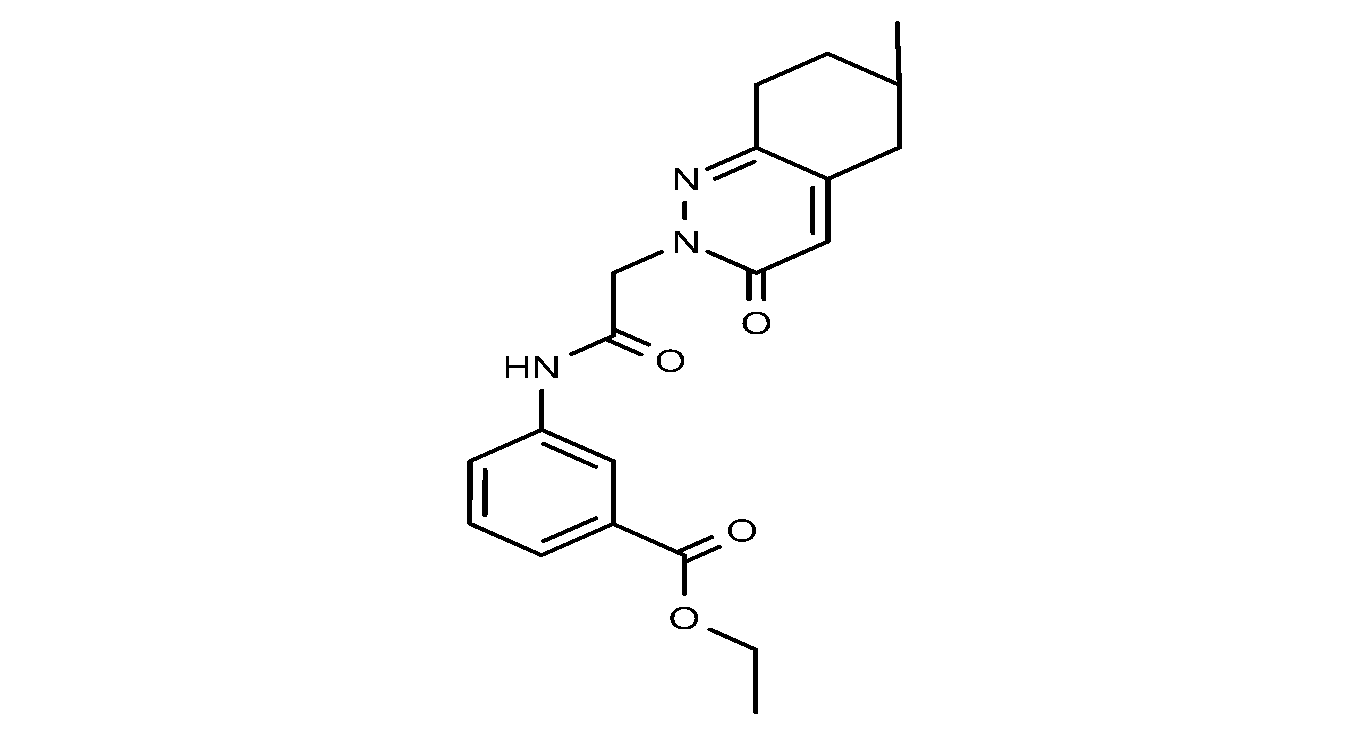 | ChemDiv | 100K | 23 |
| 4 | PCM-0070991 | 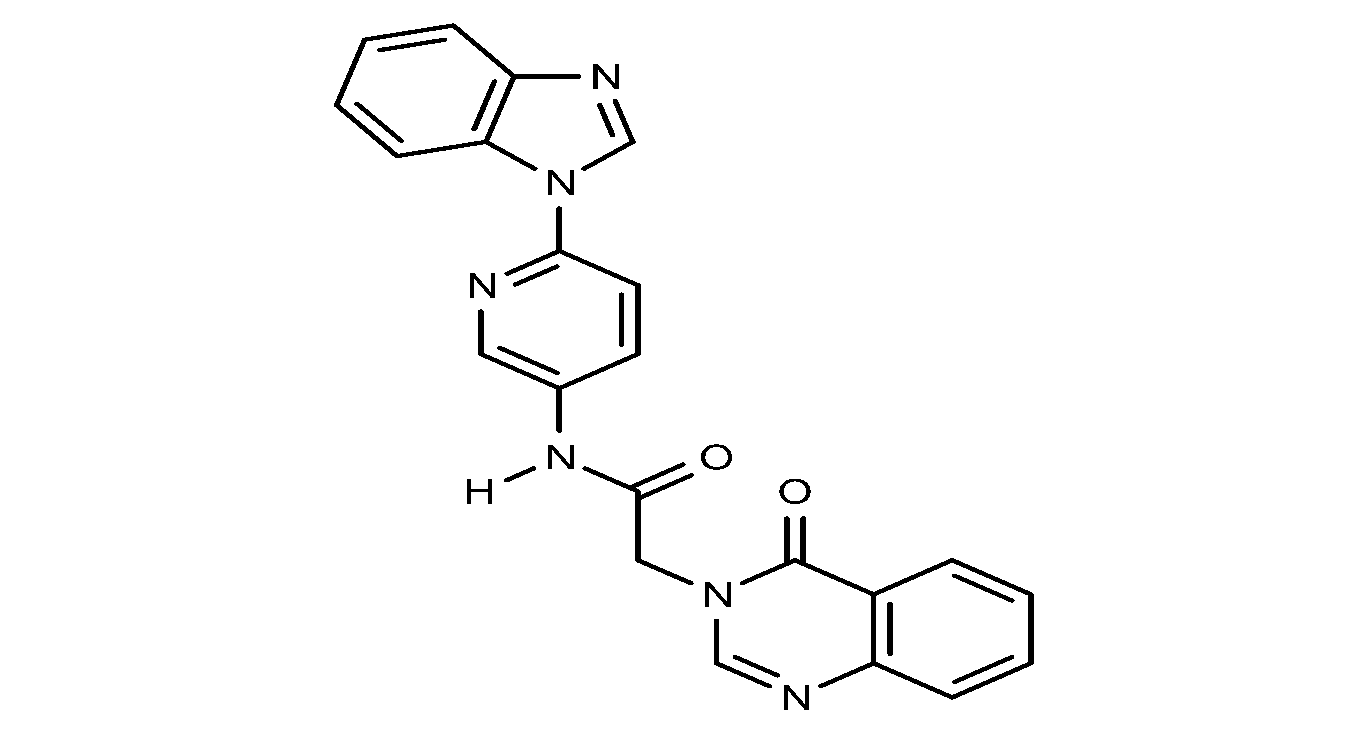 | Enamine | DLS | 12 |
| 5 | PCM-0070592 | 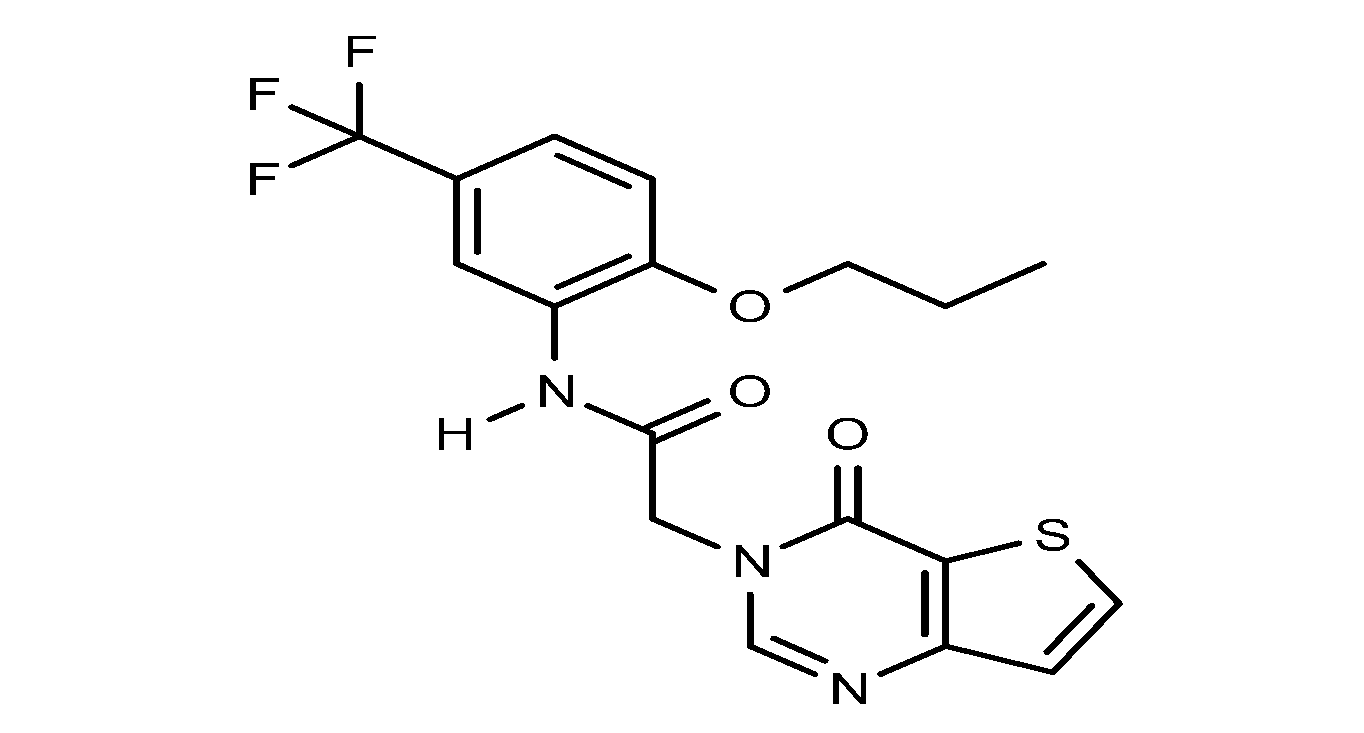 | Enamine | DLS | 30 |
| 6 | PCM-0068526 | 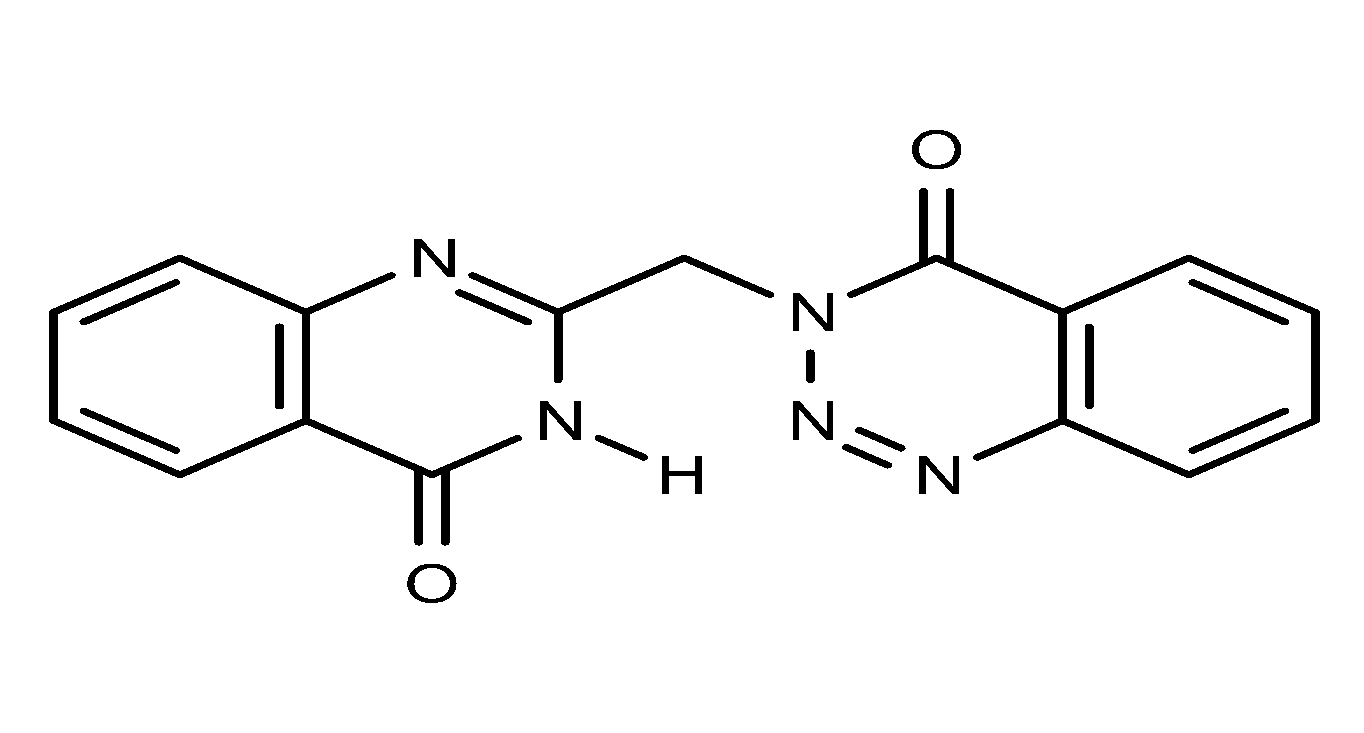 | Enamine | DLS | 30 |
| 7 | PCM-0001960 | 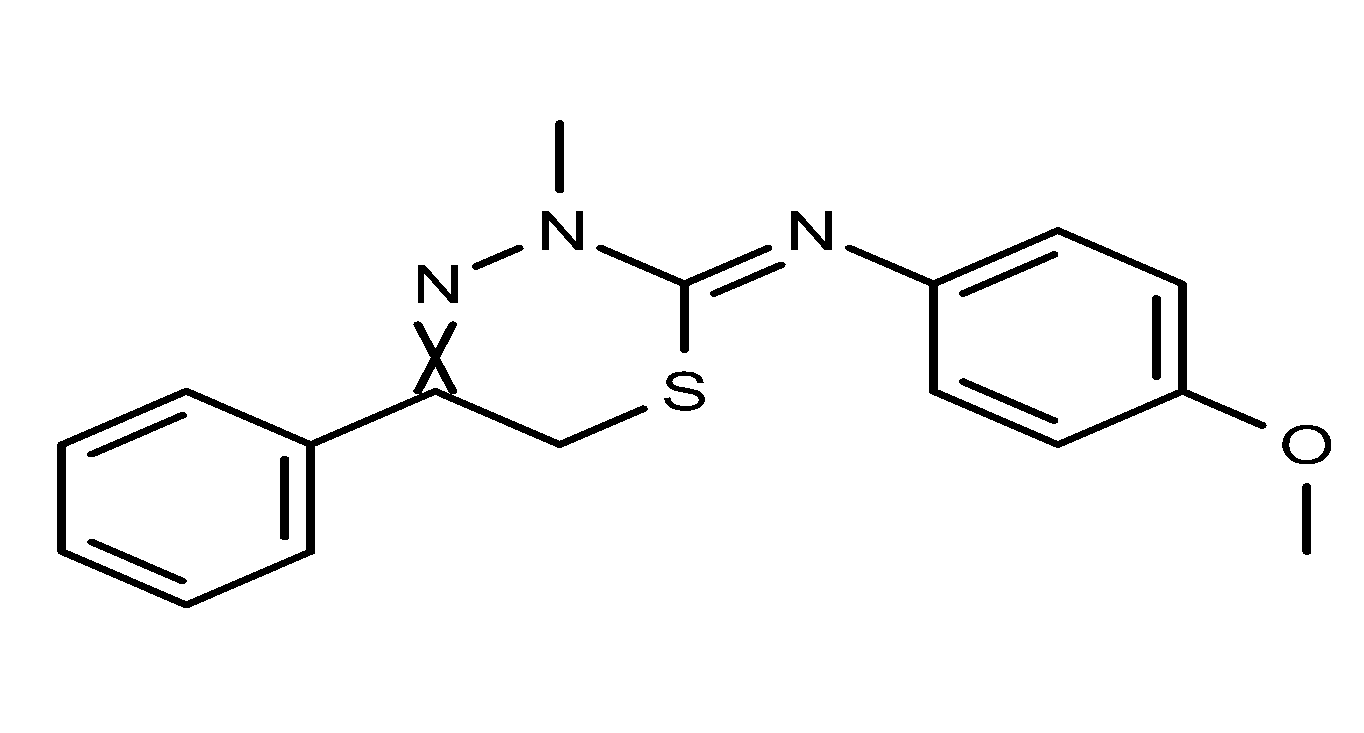 | MayBridge | HitFinder | 38 |
| 8 | PCM-0002433 | 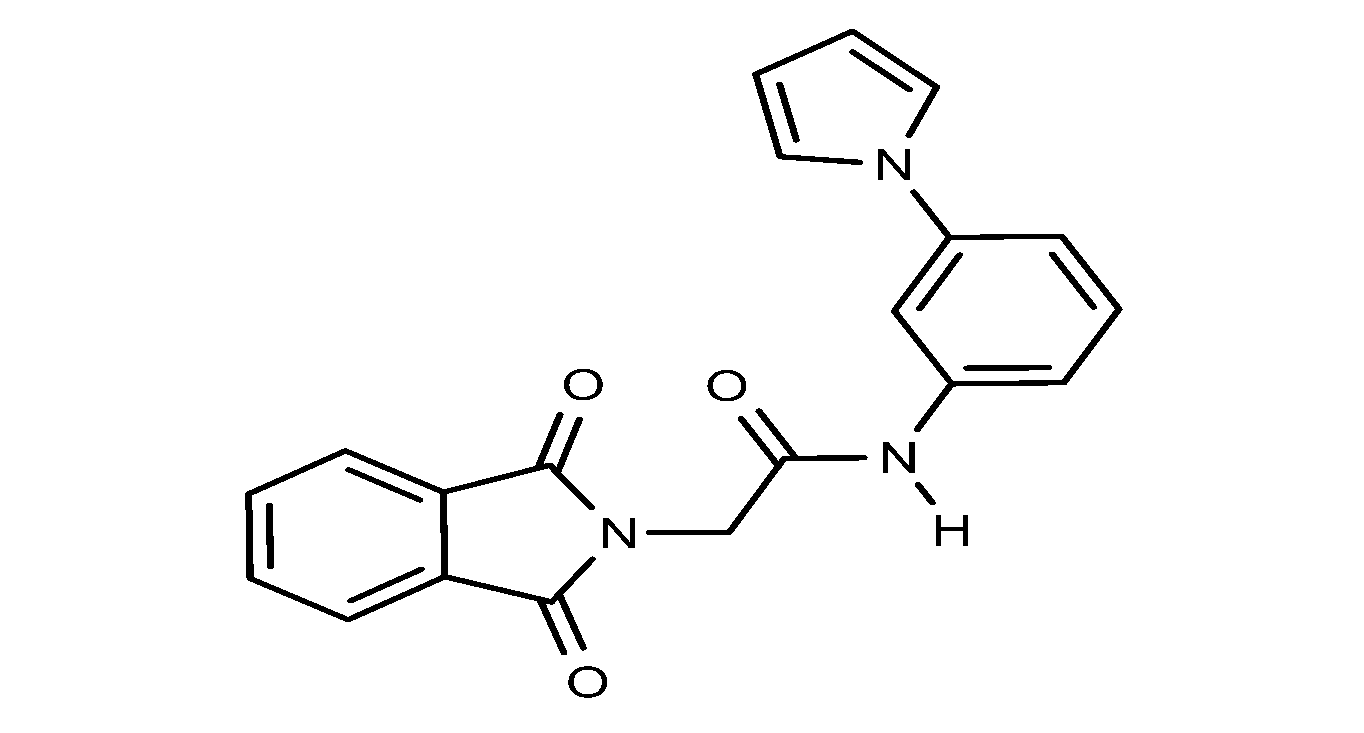 | MayBridge | HitFinder | 9 |
